# Synthesis, Crystal Structures and Photoluminescent Properties of One-Dimensional Europium(III)- and Terbium(III)-Glutarate Coordination Polymers, and Their Applications for the Sensing of Fe^3+^ and Nitroaromatics

**DOI:** 10.3389/fchem.2019.00728

**Published:** 2019-11-05

**Authors:** Sajjad Hussain, Xuenian Chen, William T. A. Harrison, Mark R. J. Elsegood, Saeed Ahmad, Shujun Li, Shabbir Muhammad, David Awoyelu

**Affiliations:** ^1^Henan Key Laboratory of Boron Chemistry and Advanced Energy Materials, School of Chemistry and Chemical Engineering, Henan Normal University, Xinxiang, China; ^2^Department of Chemistry, Mohi-Ud-Din Islamic University, Azad Jammu and Kashmir, Pakistan; ^3^College of Chemistry and Molecular Engineering, Zhengzhou University, Zhengzhou, China; ^4^Department of Chemistry, University of Aberdeen, Aberdeen, United Kingdom; ^5^Chemistry Department, Loughborough University, Loughborough, United Kingdom; ^6^Department of Chemistry, College of Sciences and Humanities, Prince Sattam Bin Abdulaziz University, Al-Kharj, Saudi Arabia; ^7^Department of Physics, College of Science, King Khalid University, Abha, Saudi Arabia

**Keywords:** europium(III), terbium(III), glutarate, X-ray structure, luminescence, sensors

## Abstract

Two lanthanide–glutarate coordination polymers, *viz*. : {[Eu(C_5_H_6_O_4_)(H_2_O)_4_]Cl}_*n*_, (**1**) and [Tb(C_5_H_7_O_4_)(C_5_H_6_O_4_)(H_2_O)_2_]_*n*_, (**2**) have been synthesized and characterized by IR spectroscopy, thermogravimetric analysis, and X-ray crystallography. In **1**, the Eu(III) ions are coordinated by four O atoms from two bidentate chelating carboxylate groups, one O atom from a bridging carboxylate group and four O atoms from water molecules adopting an EuO_9_ distorted tri-capped trigonal prismatic coordination geometry. In **2**, the Tb(III) ions are coordinated by six O atoms from three bidentate chelating carboxylates, one O atom from a bridging carboxylate and two O atoms from water molecules to generate distorted tri-capped trigonal prismatic TbO_9_ polyhedron. In both compounds, the metal polyhedra share edges, producing centrosymmetric Ln_2_O_2_ diamonds, and are linked into [001] chains by bridging glutarate di-anions. The crystal structures are consolidated by O–H···O and O–H···Cl hydrogen bonds in **1**, and O–H···O hydrogen bonds in **2**. Compound **1** exhibits a red emission attributed to the ^5^D_0_ → ^7^F_*J*_ (*J* = 1–4) transitions of the Eu(III) ion, whereas **2** displays green emission corresponding to the ^5^D_4_ → ^7^F_*J*_ (*J* = 0–6) transitions of the Tb(III) ion. Both the compounds exhibit high sensitivity and selectivity for Fe^3+^ ions due to luminescence quenching compared with other metal ions, which include; Na^+^, Mg^2+^, Al^3+^, Cr^3+^, Mn^2+^, Fe^2+^, Co^2+^, Ni^2+^, Zn^2+^ and Cd^2+^. Compounds **1** and **2** also show high luminescence quenching sensitivity for 4-nitrophenol over the other aromatic and nitroaromatic compounds, namely; bromobenzene, 1,3-dimethylbenzene, nitrobenzene, 4-nitrotolune, 4-nitrophenol, 2,6-dinitrophenol and 2,4,6-trinitrophenol.

## Introduction

Lanthanide coordination compounds have attracted great interest from the scientific community in the last two decades due to their potential as an emerging type of multifunctional luminescent materials in areas such as telecommunications, optical amplifiers, immunoassays, and sensors (Werts, 2005; Binnemans, 2009; Armelao et al., 2010; Bünzli, 2010, 2015; Zhang et al., 2010; Azab et al., 2013; Feng and Zhang, 2013; Heffern et al., 2013; Xiang et al., 2017). They exhibit unique optical properties, such as large Stokes, shift, characteristic narrow line-like emission bands, and long lived excited state lifetimes resulting from intra configurational 4*f* −4*f* transitions (Werts, 2005; Allendorf et al., 2009; Armelao et al., 2010; Bünzli, 2010, 2015; Feng and Zhang, 2013; Heffern et al., 2013; Räsänen et al., 2014; Xiang et al., 2017). The luminescent nature of lanthanide coordination compounds is associated with the organic ligand moieties and lanthanide centers (Xiang et al., 2017). Due to the low molar extinction coefficient of the Laporte forbidden *f*-*f* transitions, the direct photoexcitation of the lanthanide ions becomes difficult (Werts, 2005; Armelao et al., 2010; Bünzli, 2010, 2015; Heffern et al., 2013; Xiang et al., 2017). The introduction of suitable linkers (ligands) provides an alternative pathway for energy transfer and enriches the lanthanide emitting levels. These ligands transfer energy from the excited triplet state of ligand to the lowest emitting level of the lanthanide ion. The lanthanide ion then relaxes to the ground state by emission of energy (Reinhard and Güdel, 2002; Werts, 2005; Allendorf et al., 2009; Azab et al., 2013; Heffern et al., 2013; Rao et al., 2013; Räsänen et al., 2014; Bünzli, 2015; Xiang et al., 2017). The luminescent properties are not only related to the composition of the materials, but are also heavily dependent on the structure and intermolecular packing for their energy transfer (Cui et al., 2011; Xiang et al., 2017). Choosing appropriate ligands to increase the excited state lifetime and quantum yield (the light output) of lanthanide(III) complexes is thus essential for the development of improved luminescent materials (Li and Yan, 2012). In this regard, aromatic carboxylic acids such as, terephthalic acid (Daiguebonne et al., 2008; Wang et al., 2012) benzene-carboxylic acids (Yan et al., 2005; Wang et al., 2010b, 2012; Zhuravlev et al., 2011; Gai et al., 2013), and pyridine-dicarboxylic acid (Reinhard and Güdel, 2002; Huang et al., 2008; Zhu et al., 2009; Song et al., 2016; Xiang et al., 2017; Kumar et al., 2018) have been particularly used to design luminescent lanthanide coordination polymers. A number of studies also exist on the complexes of aliphatic carboxylic acids such as, malonic acid (Hussain et al., 2019), succinic acid (Cui et al., 2005; De Oliveira et al., 2013), glutaric acid (Głowiak et al., 1987; Legendziewicz et al., 1999; Antic-Fidancev et al., 2002), and adipic acid (Wang et al., 2010b).

From the large number of lanthanide coordination polymers, only some of them have been found active and explored for luminescence changes when brought in contact with some analyte or target molecules, possessing promising application as chemical sensors (Zhao et al., 2004; Zhang et al., 2010; Azab et al., 2013; Wang et al., 2014; Xiang et al., 2017). Thus, the optical properties of lanthanides are a beneficial tool to design new lanthanide based sensors, such as sensing of temperature (Miyata et al., 2013; Rao et al., 2013), cations (Zhao et al., 2004, 2014a; Chen et al., 2009; Zhang et al., 2010; Bogale et al., 2016, 2017a), anions (Wong et al., 2006; Tan and Wang, 2011) nitroaromatics (Bogale et al., 2016, 2017a) and small molecules (Zhou et al., 2013; Zhao et al., 2014b). Such developments also encouraged us to synthesize new lanthanide coordination polymers with characteristic properties to selectively recognize and sense the specific analyte. Particularly, we are interested to develop a highly effective, quick, and reliable method for the detection of iron and nitroaromatics because of their essentiality in health care and high toxicity/explosiveness, respectively.

Among the luminescent lanthanide complexes, those of europium(III) and terbium(III) (emitting red and green light, respectively) have been especially widely studied because of their efficient luminescent properties (Cui et al., 2005; Bangaru et al., 2010; Tsaryuk et al., 2010; Zhuravlev et al., 2011; Hussain et al., 2019). To further explore the luminescent behavior of such complexes and as a part of our ongoing research on lanthanide complexes (Hussain et al., 2014, 2015a,b, 2018, 2019) herein, we report the structural characterization and photoluminescent properties of two one-dimensional Eu(III) and Tb(III) coordination polymers based on the glutarate ligand. In addition, the possible use of these complexes as sensors for detection of Fe^3+^ and 4-nitrophenol was investigated. Although the crystal structures of a number of lanthanide-glutarate complexes have been reported in the literature (Głowiak et al., 1987; Serpaggi and Férey, 1998; Legendziewicz et al., 1999; Benmerad et al., 2000; Antic-Fidancev et al., 2002; Bromant et al., 2005; Rahahlia et al., 2006; Wang et al., 2010a; Hussain et al., 2015a; Zehnder et al., 2018) the optical properties of only a few of them have been studied (Głowiak et al., 1987; Legendziewicz et al., 1999; Antic-Fidancev et al., 2002). However, the use of none of these has been explored previously for sensing purposes. Thus, the present study would be the first one describing the potential of lanthanide-glutarates for application as sensors. As the exploitation of the new synthetic methods, structures, and properties in this system is still interesting, therefore, the studies of new lanthanide complexes with glutaric acid are important from both fundamental and applied viewpoints.

## Experimental

### Materials and Measurements

The metals salts (EuCl_3_·6H_2_O, TbCl_3_·6H_2_O) and ethanol were obtained from Alfa Aesar, USA. Glutaric acid was obtained from Merck Chemical Co. Germany. All other metal salts and nitroaromatics were purchased from Sigma Aldrich, a Johnson Matthey Company. Elemental analyses were carried out on a Varion Micro Cube, Elementar, Germany. FTIR spectra were recorded using KBr pellets on a Perkin Elmer FTIR 180 spectrophotometer over the wave number range from 4,000 to 250 cm^−1^. Thermal analyses were carried out under air with a continuous heating rate of 10°C min^−1^ from room temperature to 1,000°C on thermo-gravimetric analyzer/differential scanning calorimeter model SDT Q 600 (TA Instruments, USA) by taking 10.459 mg and 8.691 mg of compound **1** and **2**, respectively. The excitation and emission spectra were measured at room temperature in steady state mode using FLS 180 spectrophotometer equipped with Xenon Arc lamp, photomultiplier detector R928P and Czerny-Turner monochromators having focal length of 300 mm. The excitation and emission were monitored between 250–400 and 400–800 nm, respectively.

### Crystal Structure Determination

The crystal data for compound **1** and **2** were collected on a Bruker Kappa Apex II CCD diffractometer using graphite-monochromated MoKα radiation (λ = 0.71073 Å) at a temperature 150 K. The structures were solved by direct methods and the structural models were completed and optimized by refinement against *F*^2^ with SHELX-2014 (Sheldrick, 2015). The aliphatic C-bound H atoms were geometrically placed and refined as riding atoms with C–H = 0.99 Å and *U*_iso_ (H) = 1.2 *U*_eq_(C). The water molecule H atoms were located in difference maps and coordinates were freely refined with the aid of mild geometrical restraints {target: O–H = 0.84 Å} and the isotropic atomic displacement parameters were fixed to 1.5 × *U*_eq_ of the parent atom. The key crystallographic data and details of the structure refinements are provided in Table S1.

### Preparation of Complexes

#### Preparation of {[Eu(C_5_H_6_O_4_)(H_2_O)_4_]Cl}_**n**_ (1)

EuCl_3_·6H_2_O (0.183 g, 0.5 mmol) was dissolved in 15 mL deionized water and glutaric acid (0.132 g, 1.0 mmol) was dissolved in 25 mL ethyl alcohol. The solutions were mixed and stirred in a round-bottomed flask at room temperature for 3 h. 1 M NaOH solution was used to adjust pH 5–6 of the reaction mixture during stirring. The reaction mixture was filtered and left at room temperature for crystallization. Colorless block shape crystals of **1** appeared after 20 days. The crystals were recovered by vacuum filtration, rinsing with ethanol and drying in air. Yield: 51%. Analysis for C_5_H_14_O_8_ClEu: calculated (%): C 15.41; H 3.62; found (%): C 15.61; H 3.58.

#### Preparation of [Tb(C_5_H_7_O_4_)(C_5_H_6_O_4_)(H_2_O)_2_]_**n**_ (2)

TbCl_3_·6H_2_O (0.189 g, 0.5 mmol) and glutaric acid (0.132 g, 1 mmol) were dissolved in 15 mL deionized water and 20 mL ethyl alcohol, respectively. The mixture was stirred at room temperature in a round bottomed flask for 4 h. 1 M NaOH solution was added to adjust pH 4–5 of the reaction mixture. The solution was filtered and left at room temperature for crystallization. After 3 weeks, colorless block shape crystals of **2** were recovered as described above. Yield: 47%. Analysis for C_10_H_17_O_10_Tb: calculated (%) C 26.33; H 3.76; found (%): C 26.45; H 3.83.

### Photoluminescence Measurements

To investigate the detection ability of **1** toward the selected metals ions; Na^+^, Mg^2+^, Al^3+^, Cr^3+^, Mn^2+^, Fe^2+^, Fe^3+^, Co^2+^, Ni^2+^, Zn^2+^ and Cd^2+^ (as chloride salts), equal volumes (130 μL of 1 × 10^−3^ M) of metal solutions were added to **1** suspended in methanol (0.3 mg/3 mL). Likewise, the metal sensing aptitude of **2** was explored by adding equal volumes (160 μL, 1 × 10^−3^ M) of these metals to methanolic suspensions of **2** (0.5 mg / 3 mL). Luminescence titration measurements were performed by stepwise addition of Fe^3+^ solution into 0.35 mg of **1** and 0.51 mg of **2** suspended in 3 mL of methanol. After ultrasonic treatment for 5 min, their emission spectra were recorded under the same conditions at room temperature.

In the same way, to study the detection ability of **1** toward selected aromatics and nitro-aromatics including; bromobenzene (BB), 1,3-dimethylbenzene (DMB), nitrobenzene (NB), 4-nitrotolune (4-NT), 4-nitrophenol (4-NP), 2,6-dinitrophenol (DNP), and 2,4,6-trinitrophenol (TNP), equal volumes (120 μL) of the aromatics and nitroaromatics (0.001 M in ethanol) were added to 0.3 mg of **1** suspended in 3 mL of methanol. Compound **2** was tested by adding 150 μL (0.001 M in ethanol) of these compounds to 0.5 mg of **2** suspended in 3 mL methanol. The luminescence titration measurements on **1** (0.3 mg/ 3 mL methanol) were done by stepwise addition (5–160 μL) of 4-nitrophenol (0.001 M in ethanol). In the same way, **2** was assessed by the gradual addition (10–250 μL) of 4-nitrophenol (0.001 M in ethanol) to 0.5 mg of **2** suspended in 3 mL of methanol.

## Results and Discussion

### Synthesis

The reaction of EuCl_3_·6H_2_O or TbCl_3_·6H_2_O with two equivalents of glutaric acid (C_5_H_8_O_4_) in the presence of NaOH in water-ethanol medium yielded the colorless crystals of {[Eu(C_5_H_6_O_4_)(H_2_O)_4_]Cl}_*n*_, (**1**) and [Tb(C_5_H_7_O_4_)(C_5_H_6_O_4_)(H_2_O)_2_]_*n*_ (**2**), respectively. The pH value of the medium seems to have a significant influence on the nature of final products. In the preparation of complex **1**, where more amount of NaOH was added as base, the ligand was fully deprotonated to produce glutarate di-anions, while in case of **2**, the lower pH resulted in the partial deprotonatation of the ligand and produced hydrogen-glutarate anion (HGlut^−^). The proposed formulae of the complexes are in accordance with the elemental analysis. The complexes were further characterized by thermal analysis and X-ray crystallography.

### IR Spectroscopy

The IR spectra of **1** and **2** are shown in Figures S1, S2, respectively. The IR spectra of both complexes are similar, each showing broad absorption bands in the region 3,400–3,100 cm^−1^ centered at 3,387 and 3,368 cm^−1^ for **1** and **2**, respectively, which correspond to the O-H stretching vibrations of water molecules. The sharp peaks at 1,696 cm^−1^ (with a shoulder at 1,646 cm^−1^) in case of **1** and at 1,695 cm^−1^ (with a shoulder at 1,640 cm^−1^) for **2**, cannot be assigned to the asym(COO) vibration as in the free acid, but represents the O-H bending vibration due to the presence of strong H-bonded water molecules (Rahahlia et al., 2007). The bands at 1,553 and 1,525 cm^−1^ for **1** and 1,555 and 1,527 cm^−1^ for **2** correspond to ν_asym_(COO) vibrations, while those at 1,436 and 1,407 cm^−1^ for **1** and 1,437 and 1,403 cm^−1^ for **2** are associated with the corresponding symmetric modes (De Oliveira et al., 2013; Hussain et al., 2018, 2019). A difference (Δν) of about 120 cm^−1^ in ν_asym_ (CO_2_) and ν_sym_ (CO_2_) wavenumbers is indicative of the bidentate coordination of the carboxylate groups to the metal ions Eu(III) and Tb(III). For monodentate coordination of carboxylates, Δν is usually more than 200 cm^−1^ (Deacon and Philips, 1980; Zhuravlev et al., 2011; Batool et al., 2015). Monodentate coordination removes the equivalence of two oxygen atoms. If the C–O bond orders are appreciably affected, a pseudo-ester configuration is obtained. This decreases ν_sym_(CO_2_) and increases ν_asym_(CO_2_) as well as the separation (Δν) between the ν(CO_2_) values relative to those for the free carboxylate ions, usually taken as sodium salts. Chelation or symmetrical bridging should not alter the bond orders and it has been suggested that bidentate coordination gives separation similar to the ionic values. For sodium and potassium acetates, the asymm and symm modes appear at 1,578–1,571 cm^−1^ and 1,414–1,402 cm^−1^, respectively, and the respective separations are 164 and 171 cm^−1^, respectively (Deacon and Philips, 1980). Thus, the coordination mode proposed for glutarate acid in **1** and **2** is through *O, O*'-chelation of the carboxylate group. The weak bands appearing around 2,900 cm^−1^, can be assigned to the C–H stretching vibration of the glutarate ligand. The medium band around 900 cm^−1^ is ascribed to bending vibration, δ(O–C–O) of the carboxylate group (Hussain et al., 2019). The spectra also give characteristic C–C stretching vibrations of the glutaric group near 1,200 cm^−1^ (Rahahlia et al., 2007). For both **1** and **2**, the peaks in the low wavenumber region at (495, 303) and (481, 305) cm^−1^, respectively, can be attributed to weak metal–oxygen bonds.

### Thermal Analysis

Thermogravimetric analysis of both complexes reveals the loss of water molecules upon heating. The combined TGA-DSC curves of **1** are presented in Figure S3. The compound began to lose coordinated water molecules slowly at 110°C and ended at 200°C (observed weight loss 18.1%; theoretical weight loss 18.5%). The slow decomposition supports the involvement of water molecules in H-bonding. The presence of an endothermic peak at 140°C, indicates the absence of water of crystallization in agreement with the observed formula. After release of water, there is no weight loss up to 320°C, which indicates a thermally metastable product of empirical formula [Eu(C_5_H_6_O_4_)]^+^Cl^−^. Above 320°C, a continuous decomposition occurs slowly until 1,000°C releasing glutarate and chloride ions. At the end ½ Eu_2_O_3_ is left behind as a residue with an overall weight loss of 55% (theoretical 54.8%).

The thermal decomposition of **2** occurs in three stages as shown in Figure S4. At the first stage, a 7.8% weight loss occurs in the temperature range of 100 to 180°C, ascribed to the removal of two water molecules (theoretical 7.9%) followed by a plateau up to 290°C. The loss of water is associated with an endothermic transition at about 130°C. In the next stage, a 17.3% weight loss occurs between 290 and 450°C to leave probably two molecules of CO_2_ (calculated value 19.3%). The remaining fragment of one glutarate and the second ligand are released in the following step with a 35% weight loss in the temperature range of 400–800°C. Some of the oxygen atoms are used in the formation of terbium oxide. The decomposition is completed at about 800°C. The overall weight loss of 60% is in excellent agreement with the theoretical weight loss of 59.9% to leave behind ¼ Tb_4_O_7_ as a residue, which is in good agreement with that described in the literature (Cui et al., 2005). The TG results show that complex **1** possesses higher thermal stability than **2**.

### Crystal Structures

**Figure 1:**
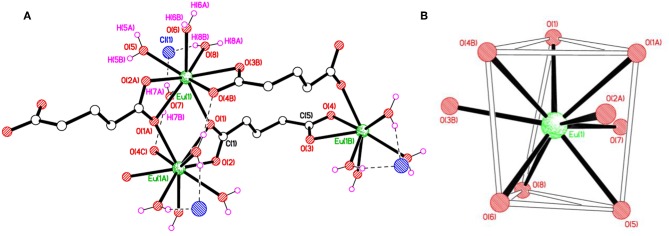
**(A)** Polymer sub-structure of {Eu(C_5_H_6_O_4_)(H_2_O)_4_]·Cl}_*n*_, **1** with the hydrogen bonds represented by dashed lines. **(B)** Distorted tri-capped trigonal prismatic coordination environment of Eu(1) in **1**.

As depicted in Figure S5, the asymmetric unit of **1** contains a Eu(III) cation, one completely deprotonated gultarate ligand, four coordinated water molecules and a chloride counter ion. Selected bond lengths and angles concerning the coordination are given in Table 1. The complex is polymeric and a segment of the polymeric structure of **1** is shown in Figure 1A. Each Eu(III) ion is coordinated by nine oxygen atoms (five from three independent glutarate ligands and four from water molecules) to form an EuO_9_ distorted tri-capped trigonal prismatic polyhedron around Eu(III) (Figure 1B). A pair of europium(III) ions are joined to each other by carboxylate oxygen (O1) atoms of two different glutarate di-anions. The metal-metal separation [Eu1···Eu1^i^ (i = 1–*x*, 1–*y*, 2–*z*)] in each pair is 4.1074 (3) Å. The dinuclear units are extended in the form of infinite [001] one dimensional chains. A polyhedral view of the chains is shown in Figure S6. The glutarate ligands exhibit identical bidentate chelating and chelating-bridging binding modes (μ_3_-κ^2^*O*:κ*O*′:κ*O*″:κ*O*^‴^). The C5/O3/O4 carboxyl group adopts a simple chelating bonding mode to a single europium(III) ion (both C–O distances are equal). The other carboxylate side (C1/O1/O2) of glutarate di-anion chelates to one metal ion and bridges to an adjacent metal ion from O1 (C1–O1 and C1–O2 bond lengths are unequal). The aliphatic chain of the glutarate is characterized by C1–C2–C3–C4 and C2–C3–C4–C5 torsion angles of −169.2 (2)° and 68.9 (3)°, respectively, i.e., a *trans*–*gauche* conformation. The Eu–O distance ranges from 2.3943(17) to 2.5217(16) Å and the average value is 2.455 Å. These distances are comparable to those in the reported nine-coordinated Eu(III)-carboxylate complexes (Cui et al., 2005; Manna et al., 2006; Zhang et al., 2007; Hussain et al., 2019).

**Table 1:** Selected bond lengths (Å) and angles (°) for complex **1**.

**Bond**	**Distance (Å)**	**Bond**	**Angle(**°**)**
Eu1—O1	2.4450 (16)	O1—Eu1—O3^i^	76.53 (5)
Eu1—O1^i^	2.4879 (16)	O1^ii^–Eu1—O3^i^	128.88 (5)
Eu1—O2^ii^	2.5217 (16)	O1—Eu1—O4i	72.85 (5)
Eu1—O4^i^	2.4494 (16)	O1—Eu1—O8	103.27 (6)
Eu1—O3^i^	2.4891 (16)	O4^i^–Eu1—O3^i^	52.70 (5)
Eu1—O5	2.4196 (17)	O5—Eu1—O2^ii^	73.53 (6)
Eu1—O6	2.3943 (17)	O5—Eu1—O7	69.77 (6)
Eu1—O7	2.4427 (18)	O6—Eu1—O5	72.75 (6)
Eu1—O8	2.4450 (17)	O6—Eu1—O1	149.26 (6)
Eu1—Eu1^ii^	4.1074 (3)	O6—Eu1—O2^ii^	74.76 (5)
O1—C1	1.288 (3)	O6—Eu1—O7	136.59 (6)
O2—C1	1.253 (3)	O7—Eu1—O8	72.98 (6)
C5—O4	1.270 (3)	O8—Eu1—O3^i^	73.18 (5)
C5—O3	1.270 (3)		

*Symmetry code: (i) –x + 1, –y + 1, –z + 1; (ii) –x + 1, –y + 1, –z + 2*.

**Figure 2:**
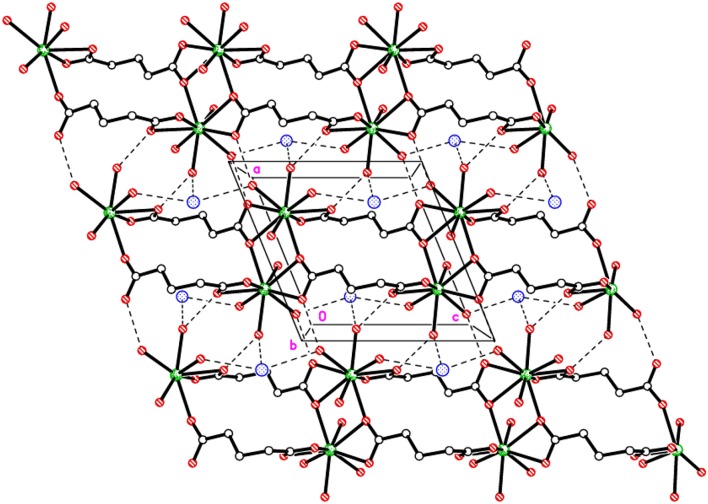
Packing plot of {Eu(C_5_H_6_O_4_)(H_2_O)_4_]·Cl}_*n*_, **1** viewed parallel to *b* showing 1D covalent ladders linked via H-bonds to form a 2D sheet.

Extensive hydrogen bonding involving coordinated water molecules and Cl^−^ ions adds stability to the coordination polymer by connecting three adjacent metal centers as shown in Figure 2. The chloride ion forms hydrogen bonds (O–H···Cl) with five water molecules. Moreover, aqua ligands act as donors to the carboxylic O atoms O2, O3, and O4 as acceptors (O–H···O). The details of hydrogen bonds are provided in Table S2. The hydrogen bonds crosslink the 1D covalent polymeric ladders into a two-dimensional sheet structure. The chloride ions in **1** occupy the regions between the (010) sheets and overall, a H-bonded three-dimensional network arises.

Compound **1** is isostructural with our previously reported neodymium analog, {[Nd(C_5_H_6_O_4_)(H_2_O)_4_]Cl}_*n*_. (Hussain et al., 2015a). In the closely related hydrated compounds {[Nd(C_5_H_6_O_4_)(H_2_O)_4_]Cl·2H_2_O}_*n*_ (Legendziewicz et al., 1999) and {[Ce(C_5_H_6_O_4_)(H_2_O)_4_]Cl·2H_2_O}_*n*_ (Rahahlia et al., 2006) similar polymeric chains are formed incorporating both chloride ions and two uncoordinated water molecules per formula unit into the inter-chain voids. However, the coordination mode of glutarate ligands is different in these two compounds. The metal atoms are bound by four glutarate ions. Two of the carboxyl groups exhibit the same chelating bridging mode as observed in **1** (having two contacts to the first metal and one contact to the second metal ion). The other two carboxylates bind with two metal ions as bridging ligands instead of chelating with one metal ion as in **1**. In case of the analogous succinic acid complexes, {[M(C_4_H_4_O_4_)(H_2_O)_4_]Cl·2H_2_O}_*n*_ (M = La, Ce) (Rahahlia et al., 2007), the layer-type polymeric structure is built up from infinite chains of one-edge-sharing LnO_6_(H_2_O)_4_ polyhedra (10-coordinate metal atom) and discrete chloride ions. The metal coordination consists of six oxygen atoms belonging to symmetrically equivalent succinate ligands and four aqua ligands. Only one type of carboxylate binding mode was described; the chelating bridging mode as found for (C1/O1/O2) group in **1**. The same bonding pattern of glutarate is found in [Eu(C_5_H_6_O_4_)(H_2_O)_3_]ClO_4_ (Głowiak et al., 1987). The europium ions are nine-coordinate with the coordination sphere made up of six oxygen atoms of glutarate ions and three water molecules.

**Figure 3:**
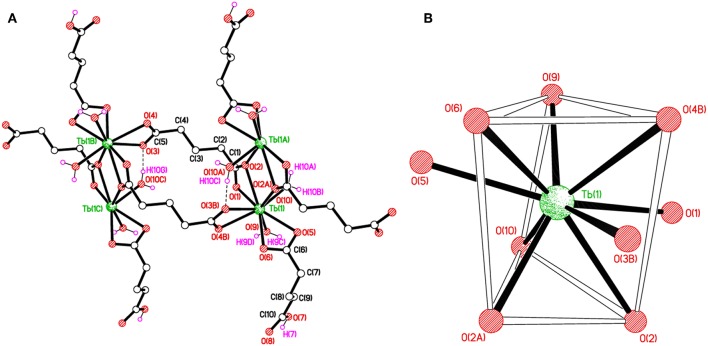
**(A)** Polymer sub-structure of [Tb(C_5_H_7_O_4_)(C_5_H_6_O_4_)(H_2_O)_2_]_*n*_, **2**. **(B)** Distorted tri-capped trigonal prismatic coordination environment of Tb(1) in **2**.

The structure of [Tb(C_5_H_7_O_4_)(C_5_H_6_O_4_)(H_2_O)_2_]_*n*_, **2** can be described as chains of dinuclear terbium(III)-(glutarate) (HGlut) units with two terbium ions linked through the O2 oxygen atoms of bridging glutarate ligands. The C6-monoanions are pendant to the chain (Figure 3A). The Tb(1)···Tb(1^i^) [(i = 1–*x*, –*y*, 1–*z*)] distance in centrosymmetric, diamond-shaped Tb_2_O_2_ units is 4.0963(5) Å. The asymmetric unit of **2** comprises of one Tb^3+^ ion, one mono-protonated glutarate ion (HGlut), one completely deprotonated gultarate dianion, and two coordinated water molecules. The selected bond parameters are listed in Table 2. Each metal ion in the polymer is coordinated by three bidentate chelating carboxylate groups (two from glutarate di-anion and one from HGlut), one bridging carboxylate O atom and two water molecules. The coordination geometry of TbO_9_ polyhedron can be described as distorted tri-capped trigonal prismatic (Figure 3B). The bond angles around the metal ions are smaller relative to those in **1** leading to a more distorted polyhedron environment for each metal center. A polyhedral view of a [001] chain in **2** showing the edge-sharing TbO_9_ polyhedra is provided in Figure S7.

**Table 2:** Selected bond lengths (Å) and angles (°) for complex **2**.

**Bond**	**Distance (Å)**	**Bond**	**Angles (**°**)**
Tb1—O1	2.448 (2)	O1—Tb1—O2	51.23 (6)
Tb1—O2	2.582 (2)	O1—Tb1—O3^ii^	91.31 (7)
Tb1—O2^i^	2.3585 (19)	O1—Tb1—O4^ii^	70.73 (7)
Tb1—O3^ii^	2.396 (2)	O1—Tb1—O5	145.14 (7)
Tb1—O4^ii^	2.542 (2)	O1—Tb1—O6	145.40 (7)
Tb1—O5	2.4706 (19)	O1—Tb1—O9	80.89 (7)
Tb1—O6	2.443 (2)	O1—Tb1—O10	77.57(7)
Tb1—O9	2.334 (2)	O2—Tb1—O2^i^	68.04 (6)
Tb1—O10	2.345 (2)	O2—Tb1—O3^ii^	74.95 (7)
C1—O1	1.256 (3)	O2—Tb1—O4^ii^	98.49 (6)
C1—O2	1.273 (3)	O5—Tb1—O6	52.86 (7)
C5—O4	1.264 (3)	O5—Tb1—O9	71.87 (7)
C5—O3	1.270 (3)	O5—Tb1—O10	76.94 (7)
C6—O5	1.269 (3)	O6—Tb1—O3^ii^	76.62 (7)
C6 —O6	1.266 (3)		

*Symmetry codes: (i) –x+1, –y, –z+1 (ii) –x+1, –y, –z+2*.

The glutarate di-anion coordinates in the same way as found in **1**. The C1-containing carboxyl group chelates to a metal ion from the C1/O1/O2 moiety and one of the chelating O atoms O(2) bridges an adjacent metal atom. The C(5)/O(3)/O(4) moiety follows a chelating bonding mode to Tb1, the next metal along the chain. The carbon-atom chain of the C(1) anion adopts an extended conformation [C1–C2–C3–C4 = 173.4(2)°; C2–C3–C4–C5 = 179.4(2)°]. The mono-anion glutarate chelates to Tb1 through C6/O5/O6 and the other end is protonated carboxylic group C10/O7/O8 does not participate in coordination. The backbone carbon chains have unique *gauche* C6–C7–C8–C9 [74.0(3)°] and *anti*. C7–C8–C9–C10 [−176.9(3)°] conformation. The mean Tb–O distance 2.434 Å is in accordance with the reported literature (Cui et al., 2005; Qiongyan et al., 2008; Hussain et al., 2019). The coordination bond lengths in **2** are slightly shorter, in comparison with **1**, which is in accordance with the slightly smaller ionic radius of Tb^3+^ (Hussain et al., 2019).

**Figure 4:**
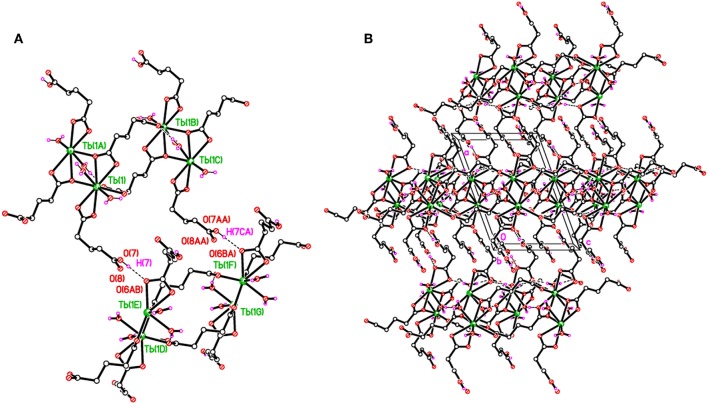
**(A)** Packing plots of **2** showing covalently-bonded 1D chains in the *c* direction. **(B)** An overall 3D H-bonded network. H-bonds shown as dashed lines.

The structure of **2** is supported by O–H···O hydrogen bonds including carboxylates and water molecules and details are given in Table S3. The carboxylate O7 hydrogen bonds crosslink the 1D polymeric chains into a two-dimensional layer as shown in Figure 4A. The layers are further connected to build a three-dimensional network (Figure 4B).

### Luminescent Properties

The solid state photoluminescence properties of **1** and **2** were investigated at room temperature.

**Figure 5:**
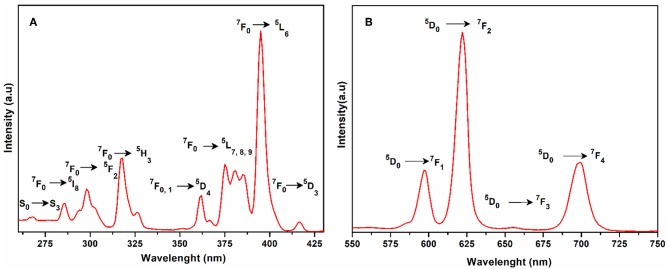
**(A)** Solid state excitation spectrum of **1** to monitor emission at 616 nm. **(B)** Solid state emission spectrum of **1** excited at 268 nm.

The excitation spectrum of **1** was measured to monitor the ^5^D_0_ → ^7^F_2_ transitions of europium at 616 nm as shown in Figure 5A. The major excitation peaks of europium are observed at 286 nm (^7^F_0_ → ^5^I_8_), 298 nm (^7^F_0_ → ^5^F_2_), 318 nm (^7^F_0_ → ^5^H_3_), 362 nm, 367 nm (^7^F_0, 1_ → ^5^D_4_), 375 nm, 381 nm, 385 nm (^7^F_0_ → ^5^L_7, 8, 9_), 395 nm (^7^F_0_ → ^5^L_6_) and 417 nm (^7^F_0_ → ^5^D_3_). All the excitation peaks in **1** arise from ground level ^7^F_0_ except ^5^D_4_ which arises from level ^7^F_1_. The most intense among them is ^7^F_0_ → ^5^L_6_. These excitation peaks have been observed in previously reported europium compounds (Baur et al., 2015; Marques et al., 2015). A very weak signal appeared at 268 nm due to transition of the ligand expected to produce luminescence emission. The other ligand transitions are superimposed on the strong ^7^F_0_ → ^5^I_8_ and ^7^F_0_ → ^5^F_2_ transition of europium. The emission spectrum of **1** was recorded under the excitation wavelength of 268 nm and is shown in Figure 5B. It exhibits four characteristic peaks in the visible region at 598, 616, 656, and 699 nm, which belong to the ^5^D_0_ → ^7^F_1_, ^5^D_0_ → ^7^F_2_, ^5^D_0_ → ^7^F_3_, and ^5^D_0_ → ^7^F_4_ transitions of the europium(III) ion, respectively. The most intense was the induced electric dipole transition ^5^D_0_ → ^7^F_2_ which is highly sensitive for the chemical bond in the environs of a europium(III) ion, and responsible for the red emission when irradiated under UV light (Cui et al., 2005; Gu and Xue, 2006; Hou et al., 2014).

Whereas ^5^D_0_ → ^7^F_1_ is the magnetic dipole transition, which is rather less sensitive to the coordinated environment and is relatively weak when compared with the electric dipole transition (^5^D_0_ → ^7^F_2_). The Eu(III) transition rule states that when the center of inversion exists on europium(III), the magnetic dipole transition (^5^D_0_ → ^7^F_1_) will be more intense emitting orange light instead of red light, emitting electric dipole transition (^5^D_0_ → ^7^F_2_) (Hou et al., 2014). The crystal field strength acting on europium(III) also affects the intensity of the magnetic dipole transition (Gu and Xue, 2006). As the site symmetry of the Eu(III) ion decreases, the intensity of the electric dipole transition ^5^D_0_ → ^7^F_2_ increases. By comparing the intensity ratio of ^5^D_0_ → ^7^F_1_ (598 nm) and ^5^D_0_ → ^7^F_2_ (616 nm) for **1**, the peak intensity ratio is about 1:3 which suggests that the coordination of the europium ions does not have an inversion center on the local site and exists in a low symmetry environment as is evident by the structural analysis of compound **1** (Hou et al., 2014).

**Figure 6:**
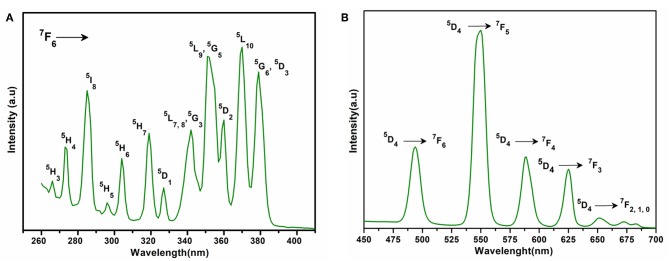
**(A)** Solid state excitation spectrum of **2** to monitor emission at 545 nm. **(B)** Solid state emission spectrum of **2** excited at 268 nm.

The excitation spectrum of **2** (Figure 6A) was measured to observe the ^5^D_4_ → ^7^F_n_ transitions of terbium at 545 nm. The excitation spectrum exhibited the following transitions of terbium; ^7^F_6_ → ^5^H_3_ (266 nm); ^7^F_6_ → ^5^H_4_ (273 nm); ^7^F_6_ → ^5^I_8_ (286 nm); ^7^F_6_ → ^5^H_5_ (296 nm); ^7^F_6_ → ^5^H_6_ (304 nm); ^7^F_6_ → ^5^H_7_ (319 nm); ^7^F_6_ → ^5^D_1_ (327 nm); ^7^F_6_ → ^5^H_7, 8_, ^5^G_3_ (342 nm); ^7^F_6_ → ^5^L_9_, ^5^G_5_ (351 nm); ^7^F_6_ → ^5^D_2_ (360 nm); ^7^F_6_ → ^5^L_10_ (370 nm); and ^7^F_6_ → ^5^G_5_, ^5^D_3_ (380 nm). All these transitions originate from the ^7^F_6_ ground state and the strongest of these are: ^7^F_6_ → ^5^L_9_, ^5^G_5_ and ^7^F_6_ → ^5^L_10_. The weak ligand transitions are superimposed on the ^7^F_6_ → ^5^H_3_ (266 nm), ^7^F_6_ → ^5^H_4_ (273 nm), ^7^F_6_ → ^5^I_8_ (286 nm), and ^7^F_6_ → ^5^H_5_ (296 nm) transition of the terbium, which are more dominant in the excitation spectra. The excitation pattern of **2** closely matches with the previously reported powder MOF of terbium-thenoyltriflouroacetone (Medina-Velazquez et al., 2019). As shown in Figure 6B under the excitation wavelength of 268 nm, the emission spectrum of **2** exhibits four intense characteristic transitions of terbium at 494 nm (^5^D_4_ → ^7^F_6_), 545 nm (^5^D_4_ → ^7^F_5_), 588 nm (^5^D_4_ → ^7^F_4_), and 625 nm (^5^D_4_ → ^7^F_3_). In spite of this, there are also three weak peaks at 652, 672, and 683 nm attributed to the ^5^D_4_ → ^7^F_2_, ^5^D_4_ → ^7^F_1_, and ^5^D_4_ → ^7^F_0_ emissions, respectively. An induced electric dipole transition (^5^D_4_ → ^7^F_5_) that is sensitive to the coordination environment of the terbium exhibits the strongest emission at 545 nm, which is characterized by the green luminescence output when the solid sample is excited under UV light. The magnetic transition (^5^D_4_ → ^7^F_6_), which is less sensitive to the coordination environment is relatively weak when compared with the electric dipole transition (^5^D_4_ → ^7^F_5_) with intensity ratio close to 1:3 (^5^D_4_ → ^7^F_6_ : ^5^D_4_ → ^7^F_5_) (Bogale et al., 2017a).

The fluorescence emissions of europium and terbium in different compounds have been reported for sensing metal ions (Zhao et al., 2004; Bogale et al., 2016, 2017a), nitro compounds (Bogale et al., 2016, 2017a), and solvent molecules (Li et al., 2013; Wang et al., 2014). The emission behavior of **1** and **2** were investigated in various solvents, such as methanol, ethanol, THF, DMSO, DMF, water, acetone, and dichloromethane. Complexes **1** and **2** show good luminescence in these solvents (Figures S8, S9, respectively) except in acetone and dichloromethane in which they exhibit a strong quenching effect on emission intensity. Thus, **1** and **2** could be used as sensors for acetone and dichloromethane. When **1** and **2** were suspended in methanol they exhibited strong emission peaks as compared with other solvents. The fluorescence life time calculated for compounds **1** and **2** are 2.499 ns and 2.895 ns, respectively.

#### Detection of Metals Ions

**Figure 7:**
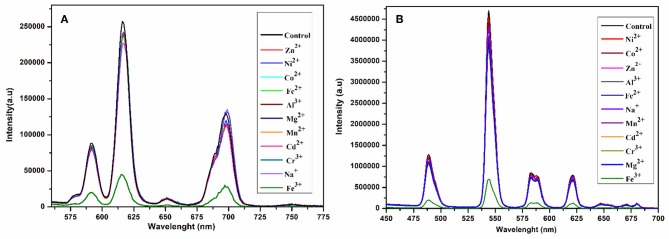
Change in emission spectra of **1** at 616 nm **(A)** and **2** at 545 nm **(B)** after interacting with different metal ions in aqueous solution under the same conditions excited at 268 nm.

**Figure 8:**
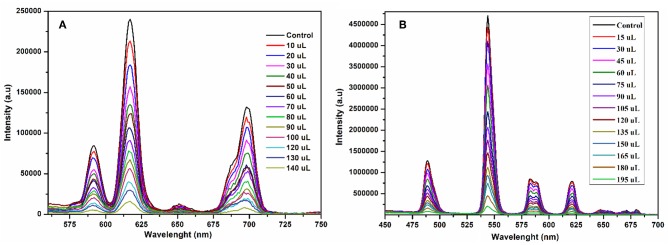
Emission spectra of **1** at 616 nm **(A)** and **2** at 545 nm **(B)** suspended in methanol upon incremental addition of Fe^3+^ in aqueous solution under the same conditions excited at 268 nm.

In order to investigate the sensing properties of complexes **1** and **2** toward various metals ions, such as Na^+^, Mg^2+^, Al^3+^, Cr^3+^, Mn^2+^, Fe^2+^, Fe^3+^, Co^2+^, Ni^2+^, Zn^2+^ and Cd^2+^, aqueous solutions of chloride salts of these metals were added individually to solutions of **1** and **2** dispersed in methanol under the same conditions. As shown in Figures 7A,B, only Fe^3+^ was able to induce significant fluorescence quenching of **1** and **2**, respectively. As compared with the initial solution (control), the fluorescence intensity of **1** and **2** were reduced about 83 and 85%, respectively, by the addition of aqueous solution of Fe^3+^, while the addition of other metals has no appreciable effect on the fluorescence intensity under the same test conditions as shown in Figures S10, S11, respectively. These results suggest that **1** and **2** are highly sensitive for the Fe^3+^ ion and induce distinct fluorescence quenching as compared with the other metals ions. Furthermore, the sensitivity tests of **1** and **2** were carried out by progressive addition of an aqueous solution of Fe^3+^ ion to the solutions of **1** and **2** suspended in methanol. The gradual addition of an aqueous solution of Fe^3+^ to the methanolic solution of **1** and **2** produced sequential quenching of fluorescence emission at 616 nm (Figure 8A) and 545 (Figure 8B) nm, respectively. The Figures S12, S13 depict that the luminescence quenching efficiencies and concentration of Fe^3+^ are in good agreement with linear proportion with R values of 0.9949 and 0.9734 for **1** and **2**, respectively. The detection limits for and **1** and **2**, estimated from the linear regression curves, were found to be 4.6565 × 10^−7^ M and 1.612 × 10^−7^ M, respectively, which is comparable with the reported terbium and europium coordination polymers for the detection of Fe^3+^ (Bogale et al., 2016, 2017a,b; Gao et al., 2016). The Stern-Volmer equation $\frac{I_{0}}{I}=\left(1+K_{sv}\left[Q\right]\right)$ was used; where *I*_0_ and *I* are luminescence intensity before and after addition of quencher, respectively, *Q* is the quencher concentration and *K*_*sv*_ is the quenching coefficient constant. The value of *K*_*sv*_ was found to be 1.31 × 10^5^ M^−1^ and (for **1**) and 8.07 × 10^4^ M^−1^ (for **2**), which suggest that the static and dynamic quenching processes are dominant as revealed by the reported studies (Bogale et al., 2016, 2017a,b; Gao et al., 2016).

The sensitivity and selectivity of compounds **1** and **2** toward Fe^3+^ were speculated due to the reason that Fe^3+^ ions diffused into the pores generated by the one-dimensional layers of **1** and **2**, and interact with the carboxylate oxygen and aqua ligands (Dong et al., 2015). Thus, complexation and incorporation of Fe^3+^ may reduce the energy transfer from ligand to Eu^3+^ and Tb^3+^ in **1** and **2**, respectively, and produce efficient fluorescence quenching. Another possible reason for luminescence quenching is exchange of Eu^3+^ and Tb^3+^ with Fe^3+^ in **1** and **2**, respectively (Zheng et al., 2013; Bogale et al., 2016).

### Detection of Aromatics

**Figure 9:**
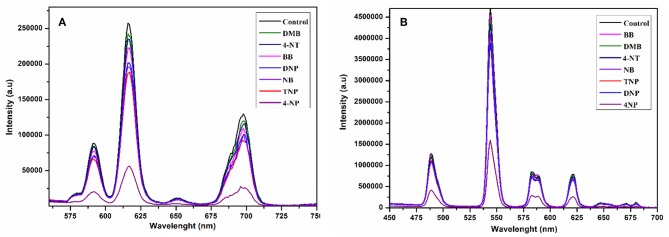
Change in emission spectra of **1** at 616 nm **(A)** and **2** at 545 nm **(B)** after interacting with different aromatics and nitro-aromatics in ethanol under the same conditions excited at 268 nm.

To scrutinize the detection capability of **1** toward different aromatic and nitroaromatic compounds; bromobenzene (BB), 1,3-dimethylbenzene (DMB), nitrobenzene (NB), 4-nitrotolune (4-NT), 4-nitrophenol (4-NP), 2,6-dinitrophenol (DNP), and 2,4,6-trinitrophenol (TNP) were selected. In doing so, equal volumes of these compounds were added to the methanolic suspension of **1** and their luminescence spectra were recorded as shown in Figure 9A. Interestingly, the emission intensity of **1** was highly reduced after addition of 4-nitrophenol, while the other analogous compounds have negligible quenching effect on the emission intensity. The decreasing potential for quenching efficiency is described as: 4-nitrophenol (4-NP) > 2,4,6-trinitrophenol (TNP) > nitrobenzene (NB) > 2,6-dinitrophenol (DNP) > bromobenzene (BB) > 4-nitrotolune (4 NT) > 1,3-dimethylbenzene (DMB), which deviates a little from the electron deficient trend of these compounds. As shown in Figure S14, 4-nitrophenol is the most efficient quencher for **1** with quenching efficiency of 78% as compared to the other electron deficient nitroaromatics, such as 2,4,6-trinitrophenol (27%), nitrobenzene (25%), 2,6-dinitrophenol (21%). The least quenching effect is with 1,3-dimethylbenzene (8%).

The compound **2** was also suspended in methanol to explore the sensing ability toward the above-mentioned aromatics and nitroaromatic compounds. The emission spectra were recorded after addition of aromatics and nitroaromatics into the methanolic suspension of **2**. As shown in Figure 9B, 4-nitrophenol exhibits significant fluorescence quenching as compared to the other tested compounds. The decreasing order of reducing emission intensity of **2** is 4-nitrophenol (4-NP) > 2,6-dinitrophenol (DNP) > 2,4,6-trinitrophenol (TNP) > nitrobenzene (NB) > 4-nitrotolune (4-NT) > 1,3-dimethylbenzene (DMB) > bromobenzene (BB). This trend varies from the electron deficient trend of these compounds. The maximum quenching efficiency for 4-nitrophenol (4-NP) was found to be 67.22% and the least quenching is bromobenzene, found to be 3.37%, while 2,4,6-trinitrophenol and 2,6-dinitrophenol have quenching efficiencies 14.01 and 17.44%, respectively, as shown in Figure S15.

**Figure 10:**
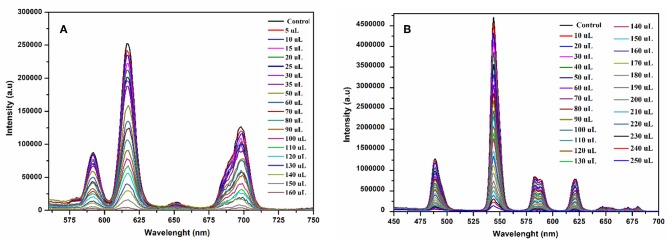
Emission spectra of **1** at 616 nm **(A)** and **2** at 545 nm **(B)** suspended in methanol upon progressive addition 4-nitrophenol ethanolic solution under the same conditions excited at 268 nm.

The fluorescence quenching titrations were performed by successive addition of 4-nitrophenol into **1** and **2** suspended in methanol. The gradual addition of 4-nitrophenol produced significant fluorescence quenching at 616 nm (for **1**, Figure 10A) and 545 nm (for **2**, Figure 10B). The Figures S16, S17 depict that the luminescence quenching efficiencies and concentration of 4-nitrophenol are in good agreement with linear proportion with *R*^2^ values of 0.9890 and 0.9888 for **1** and **2**, respectively. The detection limits for **1** and **2** from the linear regression curves were found to be 5.0589 × 10^−8^ M and 6.9173 × 10^−8^ M, respectively, which are comparable with the reported europium and terbium coordination polymers for the detection of 4-nitrophenol (Bogale et al., 2016, 2017a,b; Gao et al., 2016). The calculated Ksv values are 9.4792 × 10^4^ M^−1^ (for **1**) and 4.5295 × 10^4^ M^−1^ (for **2**), which suggest that the static and dynamic quenching processes are dominant as revealed by the previous studies (Bogale et al., 2016, 2017a,b; Gao et al., 2016).

Therefore, it is surmised that the fluorescence quenching observed in **1** and **2** by the addition of 4-nitrophenol is due to electronic interaction between 4-nitrophenol and the moieties of **1** and **2**, respectively. The other possible reasons for luminescence quenching are the inner filter effect of 4-nitrophenol and competition between absorption energy of 4-nitrophenol and excitation energy of **1** and **2** (Bogale et al., 2017b). The excitation energy absorbed by the ligands is further absorbed by the 4-nitrophenol, which is responsible for fluorescence quenching (Bogale et al., 2016).

## Conclusions

In summary, two luminescent europium(III)- and terbium(III)-glutarate coordination polymers; [Eu(C_5_H_6_O_4_)(H_2_O)_4_]Cl}_n_ (**1**) and [Tb(C_5_H_7_O_4_)(C_5_H_6_O_4_)(H_2_O)_2_]_n_ (**2**) were synthesized and characterized by IR, TGA and X-ray crystallography. The TGA curves show that after the removal of water molecules, compounds **1** and **2** are stable up to about 300°C. The photoluminescence spectra of **1** and **2** depict the characteristic peaks of europium(III) and terbium(III) ions responsible for the intense red and green emissions, respectively, when irradiated under UV light. The appearance of emission bands in the visible region in the luminescent spectra may recommend their use as long-lived luminescent probes in immuno-assays. The compounds also exhibited good luminescence in various solvents except dichloromethane and acetone. Both complexes exhibit a strong quenching effect on emission intensity when brought into contact with Fe^3+^ ions and 4-nitrophenol as compared with the other metal ions and nitroaromatics. Furthermore, fluorescence quenching titrations were performed to investigate the sensitivity of complexes for Fe^3+^ and 4-nitrophenol. By using quenching titration results, the Stern-Volmer graphs were plotted, which suggested that in all cases both static and dynamic quenching processes are dominant. The *K*_*sv*_values of **1** found for Fe^3+^ and 4-nitrophenol are 1.31 × 10^5^ M^−1^ and 9.4792 × 10^4^ M^−1^, respectively. Likewise, for 2 these values for Fe^3+^ and 4-nitrophenol are 8.07 × 10^4^ M^−1^ and 4.5295 × 10^4^ M^−1^ respectively. These results suggest that **1** and **2** can be applied as promising sensors for the detection of Fe^3+^ and 4-nitrophenol with excellent sensitivity and selectivity.

## Data Availability Statement

The datasets generated for this study are available on request to the corresponding author. CCDC numbers 1919755 and 1919756 for 1 and 2, respectively, contain the crystal data of this article. These data are available from Cambridge Crystallographic Data Center via: https://www.ccdc.cam.ac.uk/structures/.

## Author Contributions

SA and XC conceived the idea and helped in writing and proof outline. SH has a major role in the synthesis and characterization of compounds as well as in manuscript writing. ME and DA helped in data collection, structure solution, and refinement. WH wrote the crystal structure descriptions. SL and SM performed the photoluminescence related experiments.

### Conflict of Interest

The authors declare that the research was conducted in the absence of any commercial or financial relationships that could be construed as a potential conflict of interest.
