# Supplementary material for: Synthesis, Crystal Structures and Photoluminescent Properties of One-Dimensional Europium(III)- and Terbium(III)-Glutarate Coordination Polymers, and Their Applications for the Sensing of Fe3+ and Nitroaromatics
Source: Front Chem. 2019 Nov 5;7:728. doi: 10.3389/fchem.2019.00728 (PMC6849448; doi:10.3389/fchem.2019.00728)
Supplement: Supplementary file 1 [file Data_Sheet_1.pdf]

Supporting information

Synthesis, Crystal Structures and Photoluminescent Properties of One-Dimensional Europium(III)- and Terbium(III)-Glutarate Coordination Polymers, and Their Applications for the Sensing of Fe^{3+} and Nitroaromatics

Sajjad Hussain[*a,b], Xuenian Chen[*a,c], William T. A. Harrison[d], Mark R. J. Elsegood[e], Saeed Ahmad[f], Shujun Li[a], Shabbir Muhammad[g] David Awoyelu[e]

[a]*School of Chemistry and Chemical Engineering, Henan Key Laboratory of Boron Chemistry and Advanced Energy Materials, Henan Normal University, Xinxiang, 453007, China*

E-mail: xnchen@htu.edu.cn(X.C,), sajjaduet07@yahoo.com (S.H)

[b]*Department of Chemistry, Mohi-Ud-Din Islamic University, AJ&K, Pakistan*

[c]*College of Chemistry and Molecular Engineering, Zhengzhou University, Zhengzhou, China*

[d]*Department of Chemistry, University of Aberdeen, Aberdeen AB24 3UE, Scotland*

[e]*Chemistry Department, Loughborough University, Loughborough LE11 3TU, United Kingdom*

[f]*Department of Chemistry, College of Sciences and Humanities, Prince Sattam bin Abdulaziz University, Al-Kharj 11942, Saudi Arabia*

[g]*Department of Physics, College of Science, King Khalid University, Abha 61413, P.O. Box 9004, Saudi Arabia*

Table 1 Summary of crystal data and refinement details of structure refinement for compounds **1** and **2**

Compound	**1**	**2**
Formula	$C_5H_{14}ClEuO_8$	$C_{10}H_{17}O_{10}Tb$
Formula Weight	389.57	456.16
Crystal system	Monoclinic	Monoclinic
Space Group	$P2_1/c$ (No. 14)	$P2_1/c$
a, *b*, *c*, Å	8.8411(4), 15.8881(7), 8.8244(4)	12.6881(15), 13.7788(16), 8.8629(10)
β, (°)	112.2299(6)	109.9340(17)
V, $Å^3$	1147.42(9)	1456.6(3)
Z	4	4
ρ_{calc}, g cm^{-3}	2.255	2.080
μ (MoKα), mm^{-1}	5.719	4.902
F(000)	752	888
Crystal size, mm^3	0.11 × 0.09 × 0.03	0.48 × 0.02 × 0.01
Temperature, K	150(2)	150(2) K
λ, MoKα, Å	0.71073	0.71073
θ range, deg	2.49-30.55	1.71-29.64
h, *k*, *l* limits	–12:12, –22:22, –12:12	–17:17, –19:19, –12:12
Reflections, collected / unique	13630 / 3511	16229 / 4101
observed reflections [$I > 2\sigma(I)$]	3130	4101
R_{int}	0.0302	0.0434
T_{max}/T_{min}	0.847/0.572	0.953/0.202
Data / restraints / parameters	3511 / 8 / 161	4101 / 5 / 205
Goodness-of-fit on F^2	1.046	1.029
$R[F^2 > 2\sigma(F^2)]$, $wR(F^2)$	0.019, 0.041	0.024, 0.051
Largest diff. peak/hole (e Å^{-3})	0.709 /–0.746	1.055 /–0.662

Table S2. Hydrogen bond geometry parameters (Å, °) for **1**

D*—H···*A	***D*—H**	**H···*A***	***D*···*A***	***D*—H···*A***
O(5)—H(5*A*)···O(2^{iii})	0.82(2)	1.94(2)	2.755(2)	170(3)
O(5—H(5*B*)···Cl(1^{iv})	0.83(2)	2.38(2)	3.1767(18)	164(3)
O(6)—H(6*A*)···O(3^{iii})	0.81(2)	1.93(2)	2.726(2)	167(3)
O(6)—H(6*B*)···Cl(1^{v})	0.83(2)	2.29(2)	3.0985(18)	164(3)
O(7)—H(7*A*)···Cl(1)	0.81(2)	2.30(2)	3.0972(19)	167(3)
O(7)—H(7*B*)···O(4^{vi})	0.81(2)	1.88(2)	2.662(2)	165(3)
O(8)—H(8*A*)···Cl(1^{vii})	0.81(2)	2.40(2)	3.2009(19)	170(3)
O(8)—H(8*B*)···Cl(1)	0.83(2)	2.35(2)	3.1700(19)	169(3)

Symmetry code: (iii) *x*-1, *y*, *z*; (iv) *x*, -*y*+1/2, *z*+1/2; (v) -*x*, *y*+1/2, -z+3/2; (vi) *x*, *y*, *z*+1; (vii) *x*, -*y*+1/2, *z*-1/2.

Table S3. Hydrogen bond geometry parameters (Å, °) for **2.**

D*—H···*A	***D*—H**	**H···*A***	***D*···*A***	***D*—H···*A***
O(7)–H(7)···O(6^{iii})	0.81(2)	1.93(2)	2.716(3)	164(4)
O(9)–H(9C)···O(4^{iv})	0.84(2)	1.93(2)	2.774(3)	179(4)
O(9)–H(9D)···O(5^{v})	0.83(2)	1.82(2)	2.647(3)	176(4)
O(10)–H(10A)···O(3^{vi})	0.82(2)	1.87(2)	2.678(3)	167(4)
O(10)–H(10B)···O(1^{vii})	0.82(2)	1.84(2)	2.651(3)	173(4)

Symmetry codes: (iii) -x,y+1/2,-z+1/2 (iv) -x+1,y+1/2,-z+3/2 (v) x,-y+1/2,z+1/2 (vi) x,y,z-1 (vii) x,-y+1/2,z-1/2.

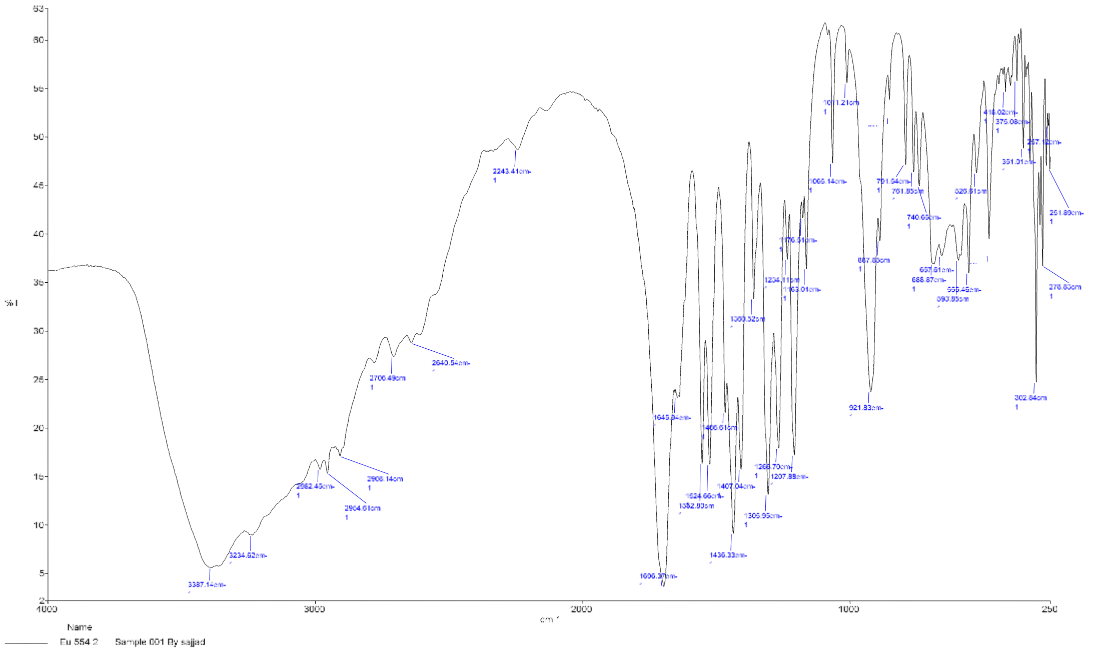

Figure S1. FTIR spectrum of $[Eu(C_5H_6O_4)(H_2O)_4]Cl$, **1**

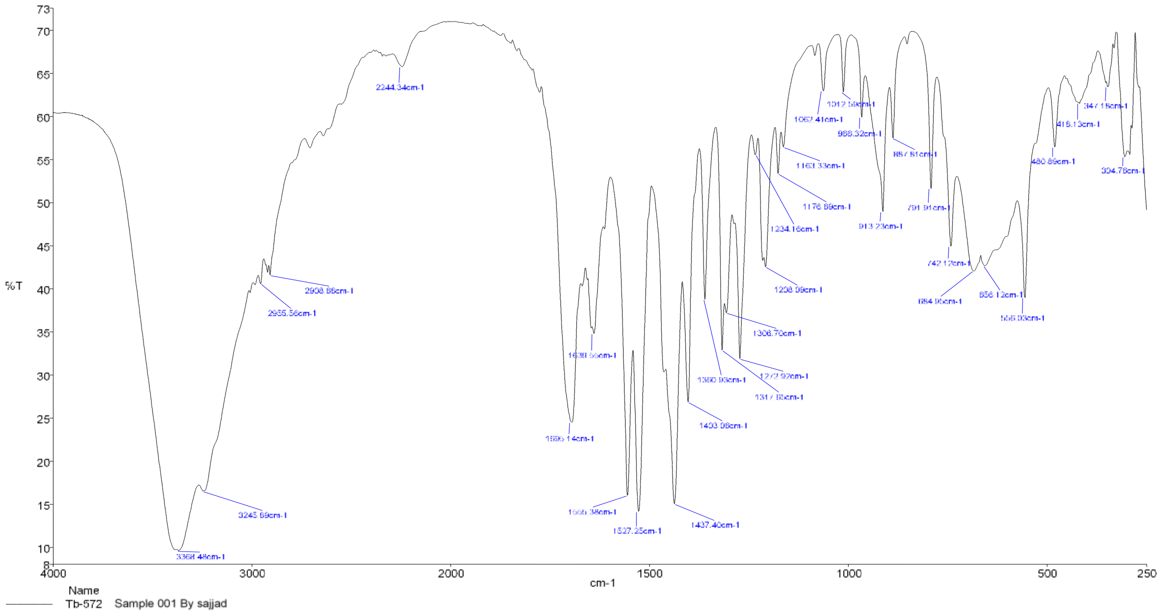

Figure S2. FTIR spectrum of $[Tb(C_5H_6O_4)(C_5H_7O_4)(H_2O)_2]_n$, **2**

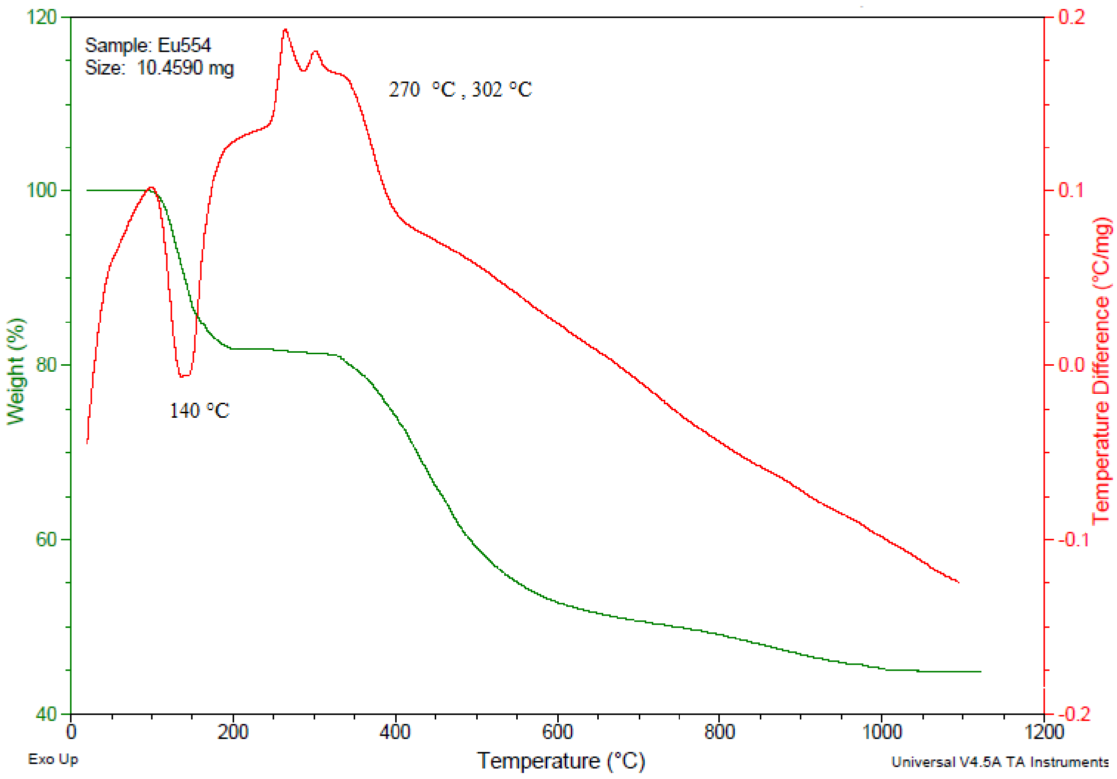

Figure S3. TGA/DSC curve of $[Eu(C_5H_6O_4)(H_2O)_4]Cl$, **1**

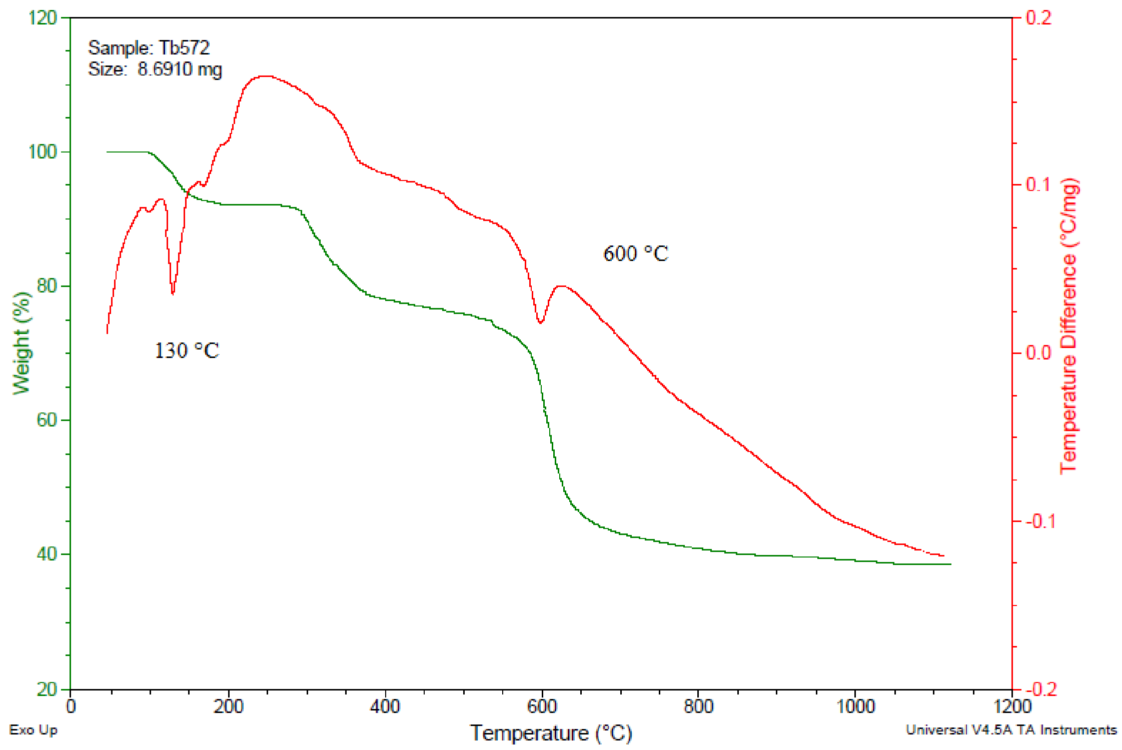

Figure S4. TGA/DSC curve of $[Tb(C_5H_6O_4)(C_5H_7O_4)(H_2O)_2]_n$, **2**

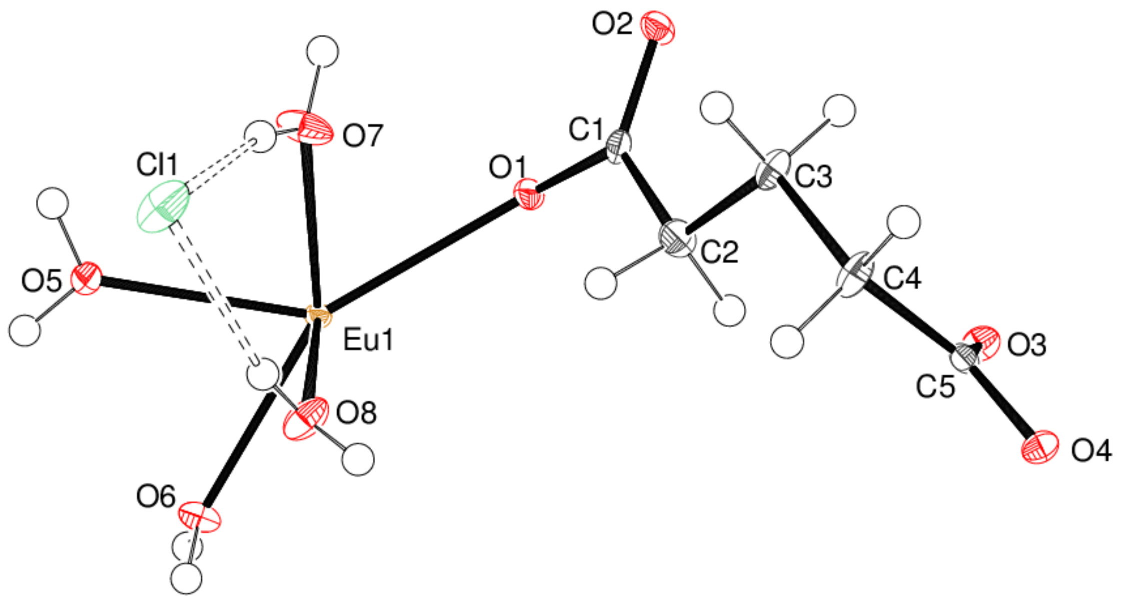

Figure S5: Asymmetric unit of $[Eu(C_5H_6O_4)(H_2O)_4]Cl$ with the O–H···Cl hydrogen bonds represented by double-dashed lines.

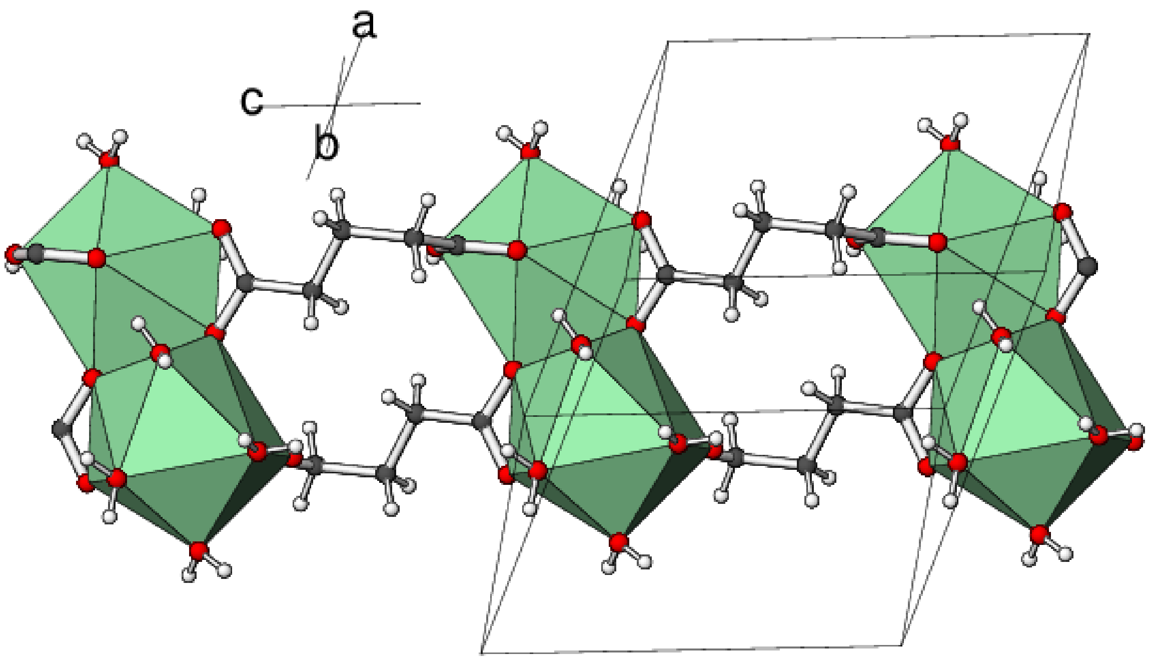

Figure S6. Polyhedral view of a [001] chain in $\{[Eu(C_5H_6O_4)(H_2O)_4]Cl\}_n$, **1** showing the edge-sharing EuO_9 polyhedra linked by the gluterate di-anions (chloride ions not shown).

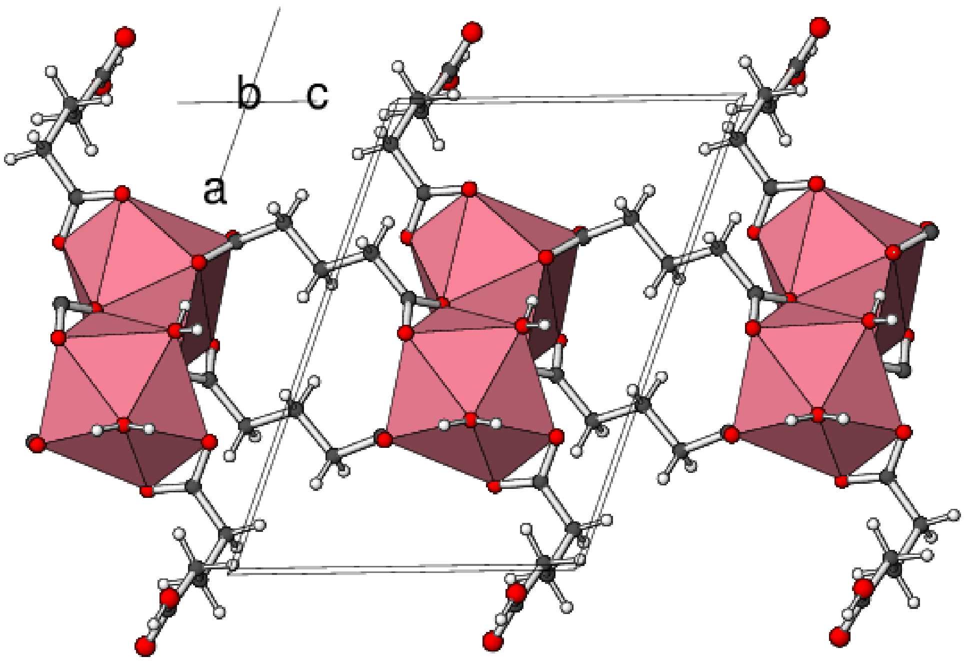

Figure S7. Polyhedral view of part of a [001] chain in $[Tb(C_5H_6O_4)(C_5H_7O_4)(H_2O)_2]_n$, **2**. The bridging glutarate dianions and the pendant hydrogen glutarate monoanions are clearly shown.

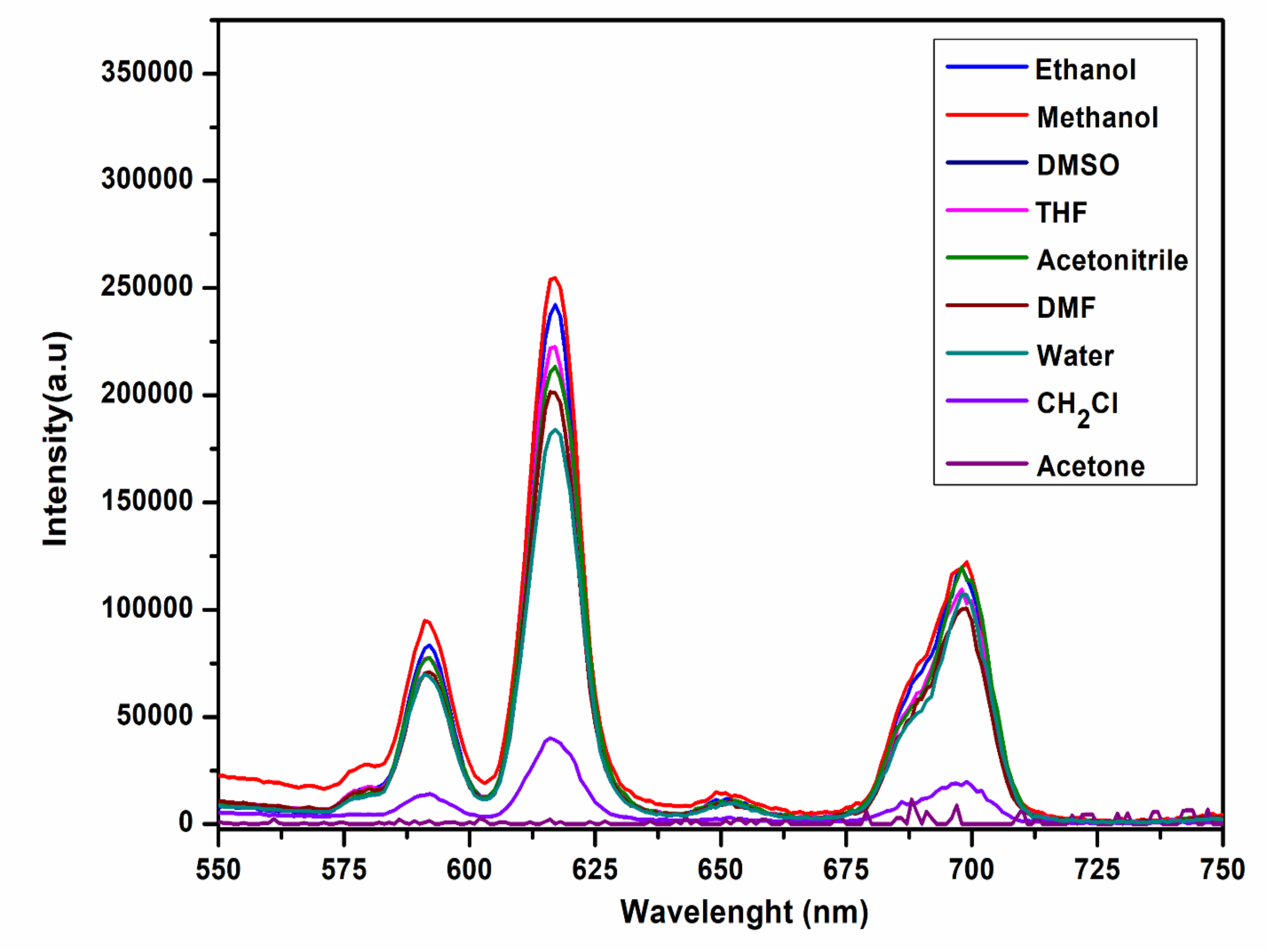

Figure S8. Emission spectra of **1** dispersed in different solvents excited at 268 nm.

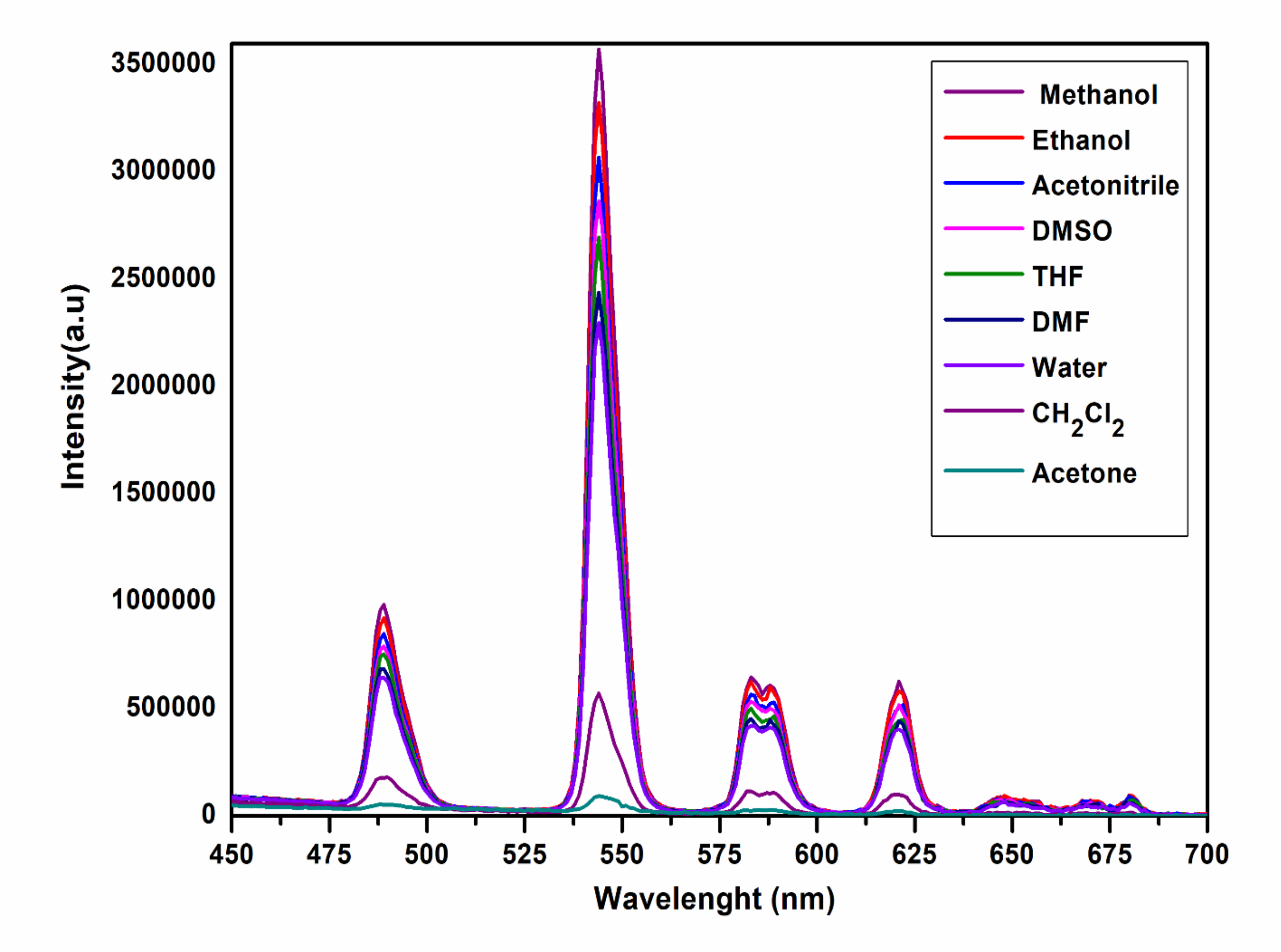

Figure S9. Emission spectra of **2** dispersed in different solvents excited at 268 nm.

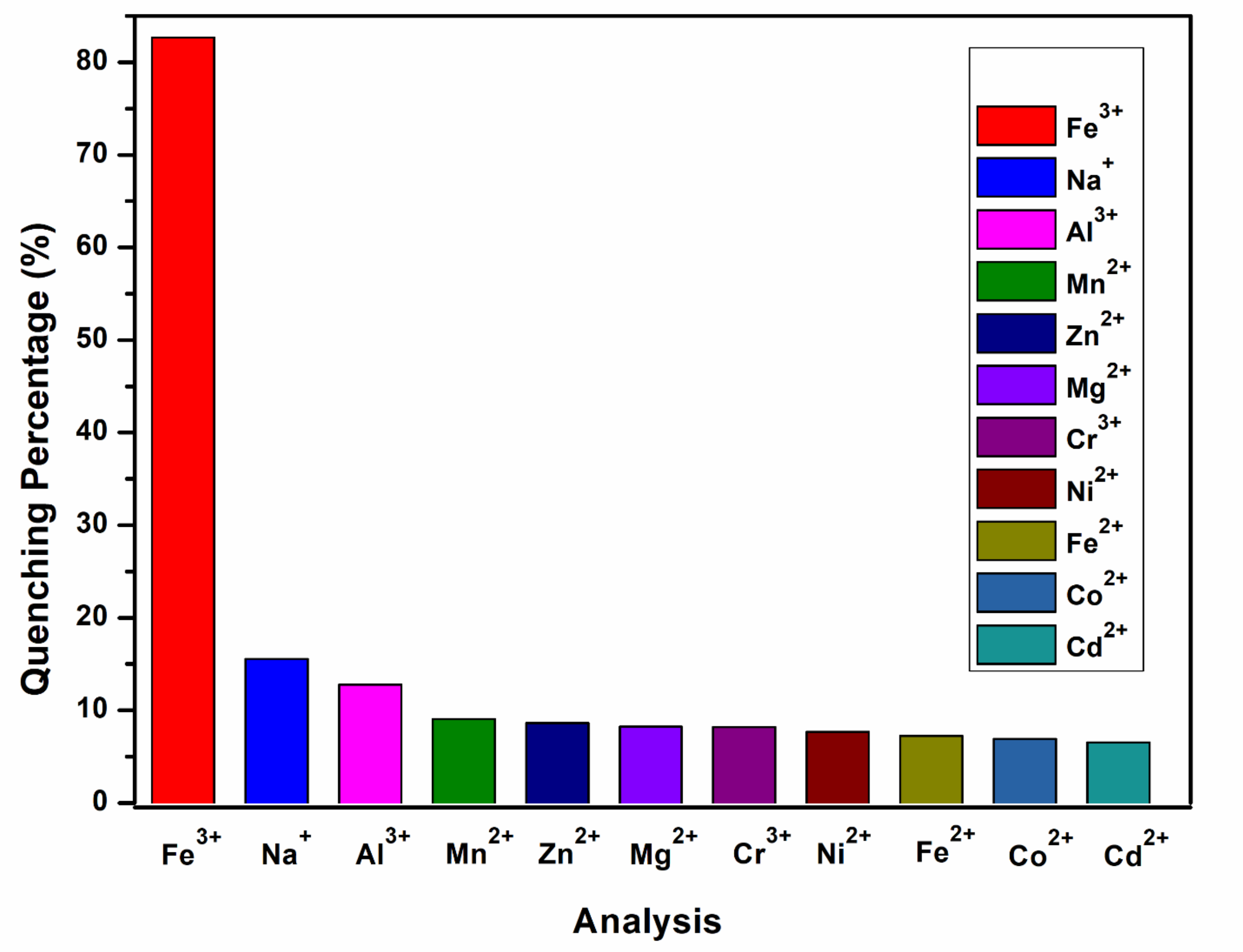

Figure S10. Percentage luminescence quenching of $[Eu(C_5H_6O_4)(H_2O)_4]Cl$, **1** towards different metals ions (excited at 268 and monitored at 616 nm)

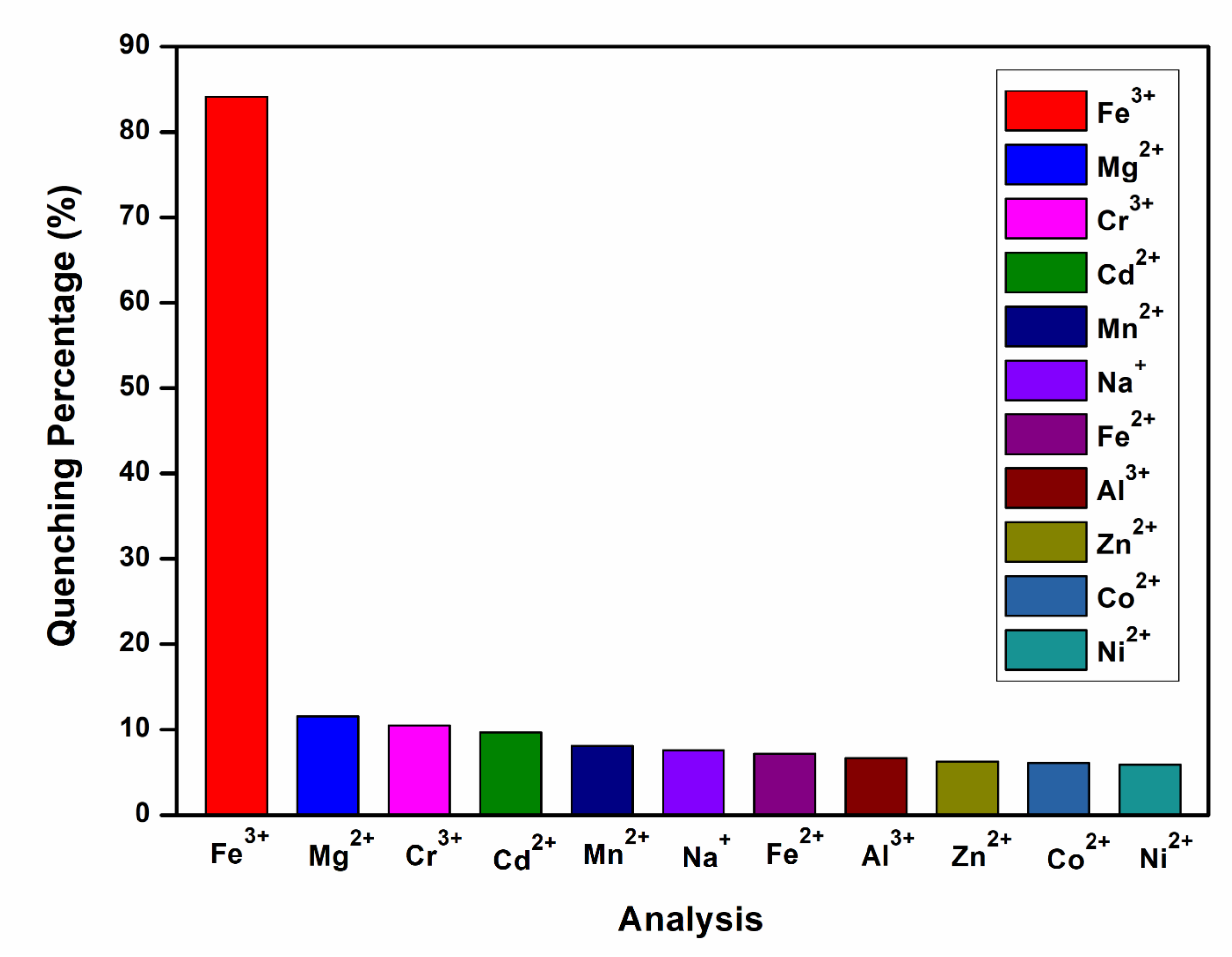

Figure S11. Percentage luminescence quenching of $[Tb(C_5H_6O_4)(C_5H_7O_4)(H_2O)_2]_n$, **2** towards different towards different metals ions (excited at 268 and monitored at 545 nm)

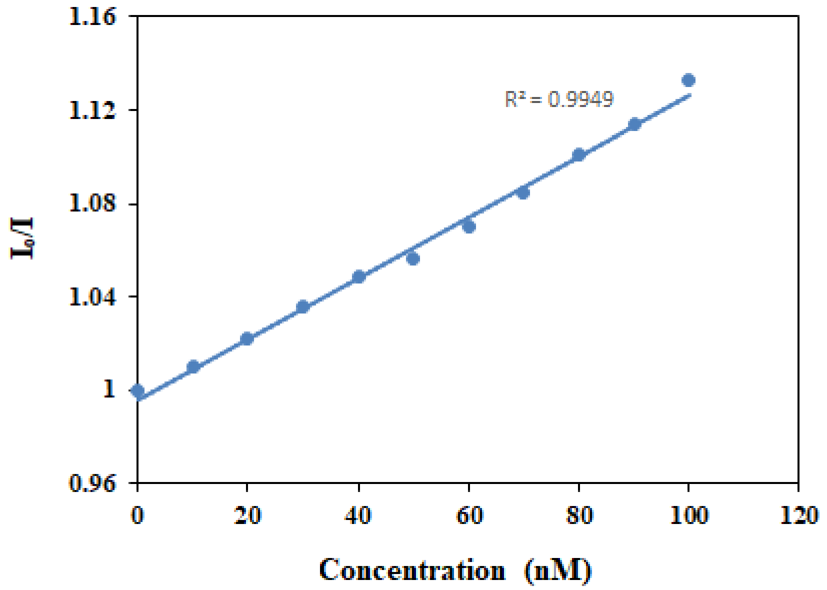

Figure S12. Stern–Volmer plot of **1** on the basis of 616 nm peak upon progressive addition of the Fe^{3+} aqueous solution.

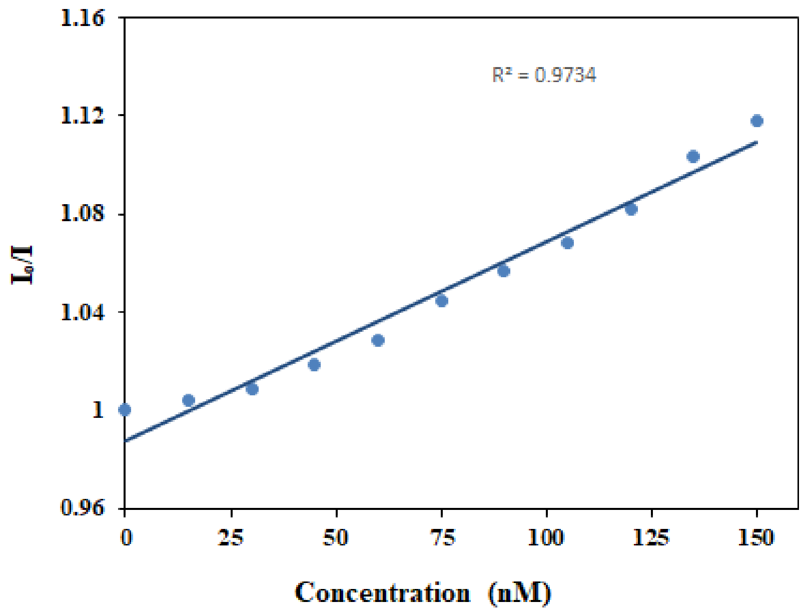

Figure S13. Stern–Volmer plot of **2** on the basis of 545 nm peak upon progressive addition of the Fe^{3+} aqueous solution

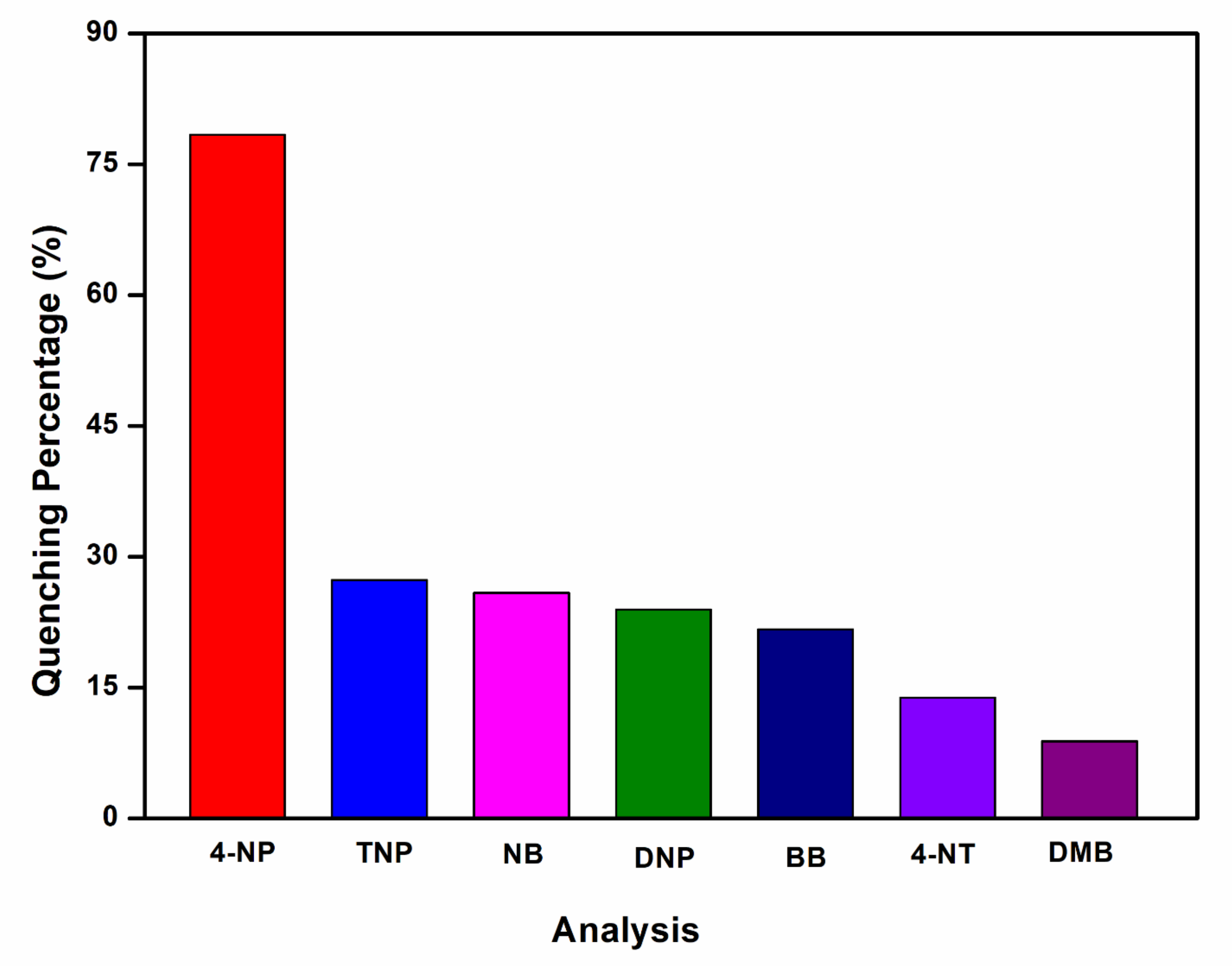

Figure S14. Percentage luminescence quenching of $[Eu(C_5H_6O_4)(H_2O)_4]Cl$, **1** towards different aromatics and nitroaromatics (excited at 268 and monitored at 616 nm)

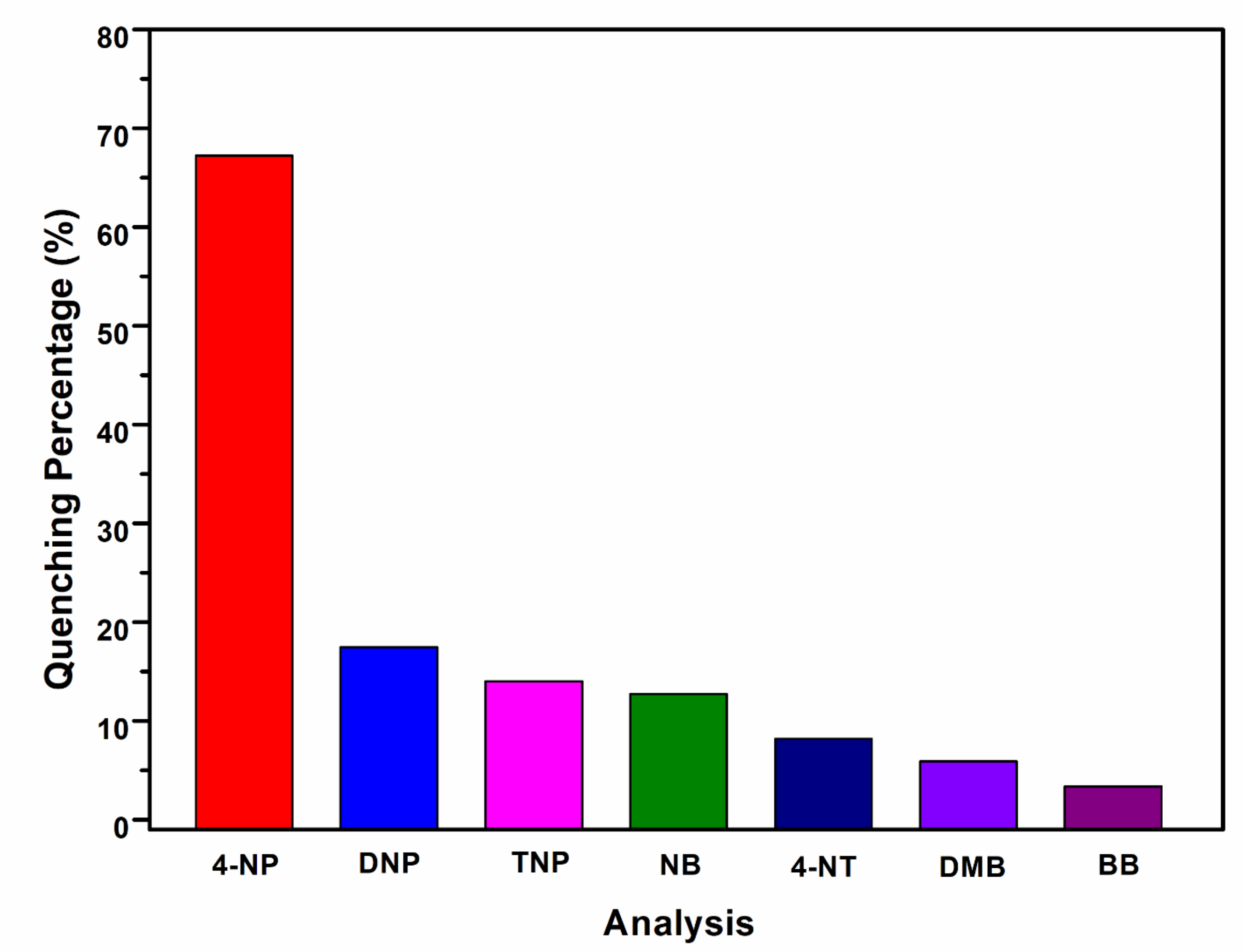

Figure S15. Percentage luminescence quenching of $[Tb(C_5H_6O_4)(C_5H_7O_4)(H_2O)_2]_n$, **2** towards different aromatics and nitroaromatics (excited at 268 and monitored at 545 nm)

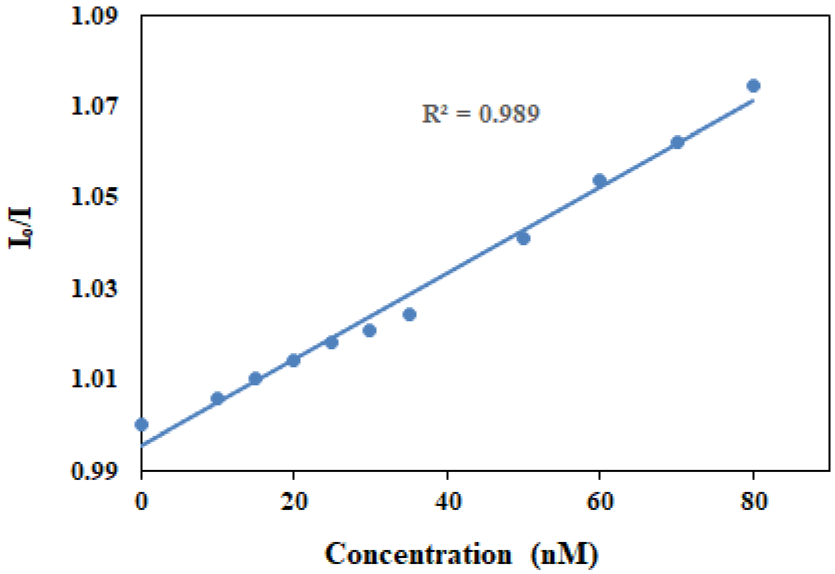

Figure S16. Stern–Volmer plot of **1** on the basis of 616 nm peak upon progressive addition of the 4-nitrophenol.

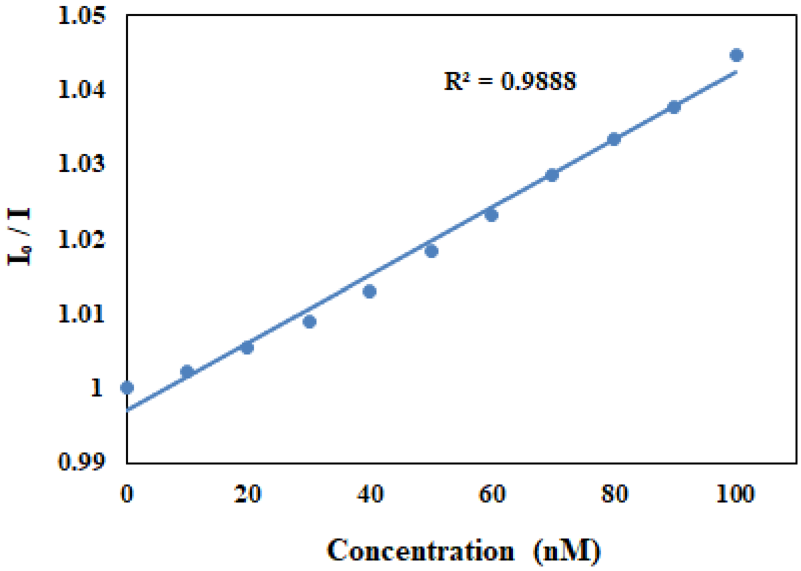

Figure S17. Stern–Volmer plot of **2** on the basis of 545 nm peak upon progressive addition of the 4-nitrophenol
